# Online Discussions of Men’s Mental Health on Reddit and YouTube: Cross-Sectional Mixed-Methods Infodemiological Study

**DOI:** 10.2196/81315

**Published:** 2026-05-05

**Authors:** Anurag Shekhar, Musawenkosi Donia Saurombe

**Affiliations:** 1 Department of Industrial Psychology and People Management College of Business and Economics University of Johannesburg Johannesburg South Africa

**Keywords:** men’s health, masculinity, mental health, social media, online peer support, emotional expression, infodemiology

## Abstract

**Background:**

Male mental health remains a major global concern, with men underrepresented in mental health care and overrepresented in suicide statistics. Masculine norms that link emotional restraint with strength can discourage help-seeking and vulnerability. Anonymous digital spaces such as Reddit (Reddit Inc) and YouTube (Google LLC) have become informal support environments where men share experiences and emotions outside traditional constraints. Understanding these interactions offers insight into masculine identity and help-seeking behavior.

**Objective:**

This study examines how men discuss and negotiate mental health within anonymous online communities. It explores whether these spaces support emotional openness, peer validation, and challenges to hegemonic masculinity norms. It triangulates digital discourse with survey and interview data to assess how these patterns align with men's lived experiences and perceived barriers to support.

**Methods:**

This cross-sectional, exploratory mixed methods study analyzed publicly available online discourse from Reddit (n=740 posts) and YouTube (n=6287 comments). The qualitative component included 23 adult men (aged 18-55 years, predominantly Asian and employed) recruited via LinkedIn (Microsoft) who completed an anonymous online survey. Of these, 9 volunteered for follow-up semistructured interviews. Data underwent computational text mining using the Natural Language Toolkit and National Research Council Lexicon for word frequency and emotion analysis, followed by Braun and Clarke’s 6-phase reflexive thematic analysis. Online discourse patterns were then compared with survey and interview data. Theoretical frameworks included hegemonic masculinity, toxic positivity, and peer-support theory.

**Results:**

Four themes emerged across the datasets: (1) normalizing emotional expression, (2) mutual validation and peer support, (3) coping through humor and irony, and (4) pushback against toxic positivity and societal norms. Emotion analysis showed prominent expressions of sadness, fear, trust, and anger across the Reddit and YouTube corpus. Survey data showed that 20 of 23 (87%) respondents reported having no safe offline space to discuss mental health. Interview participants (n=9) largely confirmed digital discourse themes, though some divergence emerged regarding whether humor functioned as deflection or connection.

**Conclusions:**

This study combines large-scale analysis of online discourse with qualitative triangulation across Reddit, YouTube, surveys, and interviews. Theoretically, it extends inclusive masculinity theory into anonymous online contexts, showing how digital platforms enable men to negotiate emotional expression outside traditional masculine constraints. It introduces the concept of “digitally mediated sanctuaries” to describe online spaces where men practice vulnerability and mutual support with less social risk. From an infodemiological perspective, the findings show how mental health information and peer-support narratives circulate and gain legitimacy within male-dominated online communities. Findings can inform gender-sensitive digital mental health interventions that build on features men already use, including humor, anonymity, and peer validation. Digital peer environments may complement formal mental health services by reducing help-seeking barriers for men hesitant to access traditional care.

## Introduction

### Background

Mental health is one of the most urgent global challenges today. More than 300 million people live with depression, and anxiety disorders affect a similar number. Suicide remains a leading cause of death among young people worldwide [1-5]. The COVID-19 pandemic, ongoing wars, and climate-related uncertainty have added to the burden of psychological distress across populations [6]. While mental illness affects people of all genders, men face particular challenges in how they experience and seek help for mental health struggles.

Men are far less likely than women to seek help when distressed. At the same time, they are more likely to die by suicide or turn to substance use as a coping mechanism [7-11]. This mismatch is often linked to social expectations about how men should behave. Traits such as toughness, emotional control, and self-reliance are rewarded in many cultures, leading many men to feel ashamed to speak about their emotions or ask for help [7,9]. These expectations can become internalized and may prevent men from recognizing when they need support.

Beyond traditional masculine norms, the cultural phenomenon of “toxic positivity” makes it harder for men to speak honestly about their emotions. This refers to the belief that people should always appear upbeat and positive, even when struggling [12-14]. Social pressure to stay strong and look happy can discourage men from reaching out, especially if they already believe that vulnerability is a sign of weakness.

For men who belong to marginalized racial, ethnic, or sexual minority groups, the risks are even higher. According to minority stress theory, people who face discrimination carry an added burden that affects their mental health [15]. These men often find it even harder to speak openly or seek support in traditional settings such as the workplace, school, or community.

In response to these barriers, many men are now discussing mental health and social constraints on anonymous platforms such as Reddit. Subreddits such as *r/MaleMentalHealth* have become spaces where men share personal struggles, reflect on emotional pain, and seek advice from peers without fear of judgment. These forums allow men to express what they often suppress in real life: vulnerability, sadness, frustration, and confusion, while receiving validation from others who understand their experience. Men frequently share culturally resonant imagery exploring emotional neglect and its consequences (refer to “Findings” subheading for examples).

YouTube has also emerged as an influential platform for men’s mental health dialogue. Videos such as TED Talks (TED Conferences, LLC) on male vulnerability, emotional well-being, and societal expectations around masculinity often attract thousands of comments. These comment sections reflect strong engagement from male viewers, many of whom share their own experiences, question stereotypes, or offer support to others. These digital interactions reveal how men navigate mental health struggles and challenge dominant narratives around emotional suppression.

Infodemiology refers to the science of the distribution and determinants of information in electronic media, especially the internet, to inform public health and policy [16]. It involves analyzing digital trace data (such as social media posts, search queries, and online discussions) to understand health information–seeking behaviors, disease surveillance, and public perceptions of health issues. From an infodemiological perspective, understanding how mental health information circulates and is negotiated in these digital spaces is critical for public health.

This study examines this discourse by analyzing 740 anonymized posts from Reddit’s r/MaleMentalHealth, 6287 comments on 4 TEDx Talks related to men’s mental health on YouTube, and qualitative data from 23 men who participated in surveys and 9 follow-up interviews. Using a mixed methods design and reflexive thematic analysis, the research examines how men express distress, negotiate masculine norms, and connect with others in online and conversational spaces. By triangulating these data sources, the study provides deeper insight into how anonymous digital platforms and lived experiences together shape contemporary understandings of men’s mental health.

This study has 3 integrated objectives: a qualitative objective to identify and describe emergent themes in men’s mental health discourse on Reddit and YouTube through reflexive thematic analysis; a quantitative objective to analyze linguistic patterns and emotional tone across the combined corpus using computational text mining and emotion lexicon analysis; and a mixed methods objective to triangulate digital discourse findings with survey and interview data, examining whether online expressions of vulnerability, peer support, and norm negotiation align with men’s lived experiences and help-seeking behaviors.

### Masculinity, Mental Health, and Help-Seeking

A growing body of evidence positions men’s mental health as a public health concern, particularly due to high suicide rates and the persistent gap in help-seeking behavior [8-11]. Despite significant mental health challenges, men are considerably less likely than women to pursue professional psychological support [7,9,11]. One major explanation lies in sociocultural norms surrounding masculinity, which discourage emotional openness and reinforce the stigma around mental illness [11].

Hegemonic masculinity, a dominant cultural ideal, promotes traits such as stoicism, emotional control, and self-reliance, which discourage men from acknowledging or disclosing distress [17]. Stronger conformity to these norms, particularly among middle-aged and older men, has been linked to elevated rates of depression [18]. While traditional masculinity may grant social status and power, it also contributes to poor mental health outcomes by framing vulnerability as weakness [8].

Nondisclosure of distress is a common consequence of these norms, with men often internalizing the belief that seeking help is a failure of masculinity [19]. This silence can compound feelings of isolation and shame. Reddit and similar forums have emerged as crucial alternative spaces where men can speak more freely [20]. Studies show that depressive symptoms in men are associated with emotional suppression, and peer environments encouraging vulnerability can help reduce such tendencies [19].

The theory of hegemonic masculinity explains how dominant ideals stigmatize traits such as fear, sadness, or emotional dependence as unmanly [21]. Men are often taught to suppress these feelings, leading to shame, substance abuse, and in some cases, suicide [7]. Toxic masculinity describes how these expectations can manifest in extreme forms, such as mocking emotional expression, which undermines men’s mental well-being [7]. Scholars have argued for a redefinition of masculinity that includes emotional expression as a form of strength, not weakness [7].

Some men grow up believing their worth is tied to their productivity, independence, and emotional detachment rather than to who they are intrinsically. This belief is vividly reflected in digital spaces, where commentary and shared narratives capture men’s sense of conditional value. A widely circulated quote expressing that men are only loved conditionally based on what they provide resonates deeply in Reddit and YouTube discussions, reinforcing the idea that men must earn their emotional worth and complicating their willingness to express vulnerability or seek help.

### Anonymity and Computer-Mediated Communication

Anonymity and computer-mediated communication dynamics help explain why digital spaces enable vulnerability and norm-challenging discourse among men. According to Suler’s [22] online disinhibition effect, features such as anonymity, invisibility, and asynchronicity reduce fear of judgment and lower self-presentation pressures, allowing individuals to disclose personal experiences more freely. Similarly, Joinson [23] found that perceived anonymity in computer-mediated communication increases self-disclosure by diminishing social desirability concerns. Within male peer communities, these affordances temporarily suspend the social costs associated with violating masculine norms, creating psychological safety to articulate emotions or seek help without reputational threat [24].

Empirical research supports this process of online identity demarginalization. McKenna and Bargh [25] demonstrated that individuals with marginalized or stigmatized identities used anonymous online groups as low-risk environments to explore and affirm aspects of self that were constrained offline—often preceding greater authenticity in face-to-face contexts [25]. Similarly, De Choudhury and De [26] found that Reddit’s structural affordances, including the use of “throwaway accounts,” enabled open discussions of highly stigmatized topics such as suicidality, depression, and anxiety [26]. These findings align with this study’s observation that Reddit and YouTube comment threads act as psychological safety zones where men renegotiate emotional boundaries and experiment with alternative masculinities beyond offline constraints.

### Intersectionality and Minority Stress

Masculinity does not operate in isolation. Intersectionality theory, as developed by Crenshaw [27,28], urges researchers to consider how gender norms intersect with race, sexuality, and other identity categories. For example, Black men may face heightened pressure to appear emotionally strong, perceiving vulnerability as doubly threatening in the context of racial stereotypes [11]. Similarly, sexual minority men face a unique combination of masculine role expectations and societal stigma, increasing their psychological burden.

Meyer’s minority stress theory further explains how systemic discrimination contributes to chronic psychological strain among individuals who identify as Lesbian, Gay, Bisexual, Transgender, Queer or Questioning, and more (LGBTQ+) [15]. Concealing one’s identity or experiencing rejection from peers can result in internalized stigma and elevated risk for depression and anxiety [15,29]. Although Reddit posts are largely anonymous, themes of intersectional stress can still emerge subtly in discussions, particularly in references to race, sexuality, or social isolation. Several posts across subreddits, including r/Science, actively engaged with research-based evidence on LGBTQ+ mental health disparities, often receiving strong support from users with similar lived experiences. This demonstrates how online communities reflect and reinforce awareness of minority stress and intersectional vulnerabilities.

### Peer Support and Inclusive Masculinities in Online Spaces

Anonymity and accessibility make online peer support forums an increasingly attractive alternative to formal therapy [20,30,31]. Platforms such as Reddit enable men to engage in open emotional dialogue without fear of judgment or social consequences [32]. Prior research shows that men often favor informal help from peers or anonymous online groups over traditional mental health services [11].

Online communities, particularly those with a clear ethos of respect and empathy, foster a sense of psychological safety, where men can disclose vulnerabilities and receive affirmation [20]. These environments can reduce self-stigma by normalizing mental health struggles and offering shared narratives of hope and recovery [20,32,33]. Peer interaction on such platforms also aligns with mechanisms of universality and altruism found in group therapy contexts, where offering support to others boosts one’s own sense of efficacy and connection.

This shift is part of a broader rethinking of masculinity. Emerging research on “positive” or “inclusive” masculinities recognizes that masculinity is plural and malleable. Rather than adhering to rigid ideals, these evolving masculinities emphasize emotional literacy, empathy, and self-care. Campaigns such as Movember’s [34] “man of more words” exemplify how men are beginning to reclaim emotional expression as a strength rather than a liability. These shifts echo the American Psychological Association’s call to promote healthier models of masculinity grounded in psychological well-being [35]. However, online discourse frequently reveals the gap between these aspirational messages and lived reality, with users critiquing the performative contradiction in societal responses.

Within this framework, online peer support operates through 2 interrelated mechanisms. At the individual level, it functions as a buffering process that mitigates the emotional strain associated with restrictive masculine norms by offering validation, empathy, and normalization of emotional expression. At the collective level, it acts as a social-influence mechanism that can gradually modify normative boundaries through repeated exposure to alternative expressions of masculinity. As men encounter peers modeling vulnerability and mutual care, these interactions may incrementally foster norm flexibility, reducing the perceived social cost of emotional openness. While our cross-sectional design cannot infer causal transformation, these discursive dynamics illustrate how digital peer spaces create psychosocial conditions conducive to both immediate coping and gradual normative reorientation.

### Toxic Positivity as a Moderating Influence

The growing cultural expectation to “stay positive” or “look on the bright side” has evolved into what commentators call toxic positivity, a mindset that discourages the open expression of distress. For men, this can reinforce long-standing social norms that equate emotional control with strength and vulnerability with weakness [24,36]. While maintaining optimism can sometimes support coping, constant pressure to appear strong or grateful often invalidates genuine pain and contributes to emotional isolation. Many men, having internalized these expectations, delay seeking help until distress becomes overwhelming [37].

In digital environments, these pressures can take new forms. Social media platforms often encourage simplified motivational interactions [38]. Yet the anonymity and reach of platforms such as Reddit and YouTube can also reduce social risk, allowing men to question or reject these pressures in ways that may not feel possible offline. In communities such as r/MaleMentalHealth, participants often encourage one another to be honest, practice self-compassion, and challenge the idea that men must always appear in control. Humor and irony frequently emerge as tools that make this disclosure feel safer and more socially acceptable, helping men connect without losing face [39].

Toxic positivity, therefore, both constrains and shapes men’s emotional expression online. It moderates how peer support unfolds by influencing which forms of vulnerability are considered acceptable and how they are communicated. Recognizing this tension helps explain how men negotiate authenticity in online spaces that promote both connection and restraint.

### Beyond Binary Masculinity Models: Toward Digital Pluralism

The relationship between masculinity and men’s well-being is increasingly understood through frameworks that reject singular or static definitions of manhood. Contemporary scholarship recognizes plural masculinities—the idea that men express gender in multiple, shifting ways across different social settings and identities [21,40,41]. This pluralistic view contrasts with traditional binary models that separate “hegemonic” and “subordinate” masculinities, instead emphasizing fluidity and context. Scholars have also argued for building upon men’s strengths to promote positive masculinities that encourage empathy, help-seeking, and emotional growth [42,43].

This theoretical perspective extends meaningfully into digital environments, where technological affordances such as anonymity and audience reach enable digital pluralism—the coexistence of multiple, flexible masculinity performances within the same interactional space. Because much existing research on masculinity is based on offline contexts, digital discourse reveals distinct mechanisms that support identity fluidity. The relative anonymity of online platforms reduces the perceived social risk of vulnerability [44], allowing men to articulate emotions or uncertainties without reputational threat.

Within anonymous forums and peer networks such as Reddit and YouTube, men shift seamlessly between vulnerability, humor, stoicism, and care, forming masculinities that are situational rather than hierarchically fixed [21,43]. Humor and irony often function as socially acceptable strategies for emotional expression, helping men discuss mental health without contravening masculine display rules [39,45]. In this sense, online spaces become sites of performative negotiation, where masculine identity is continually reshaped through interaction and mutual recognition [46,47].

Thus, masculinity in digital contexts can be understood as a dynamic interplay of diverse voices negotiating authenticity, belonging, and emotional legitimacy in real time. Analyzing these online exchanges moves beyond binary or linear models of masculinity change, positioning digital discourse as a relational arena where men experiment with and normalize alternative masculinities [24,48].

Recent experimental research further supports the transformative potential of exposure to counter-normative narratives. Borinca and Gkinopoulos [49] demonstrate that heterosexual men’s endorsement of traditional masculinity norms can be experimentally reduced when exposed to minority perspectives (ie, narratives of gay men embracing nontraditional relational and domestic roles). Their findings suggest that contact with minority masculinities fosters emotional openness by lowering the perceived threat to masculine identity. Complementing this, Borinca et al [50] show that when men perceive broader societal shifts toward more inclusive gender norms, they experience less discomfort when engaging in counter-stereotypical behaviors. Together, these studies provide causal evidence that exposure to alternative or minority masculinities, precisely what occurs in online communities where diverse vulnerability narratives coexist, can facilitate gradual attitude change. This supports our interpretation that digital peer spaces are not merely reflective but also generative environments for masculine norm transformation.

### Theoretical Framework

**Figure 1:**
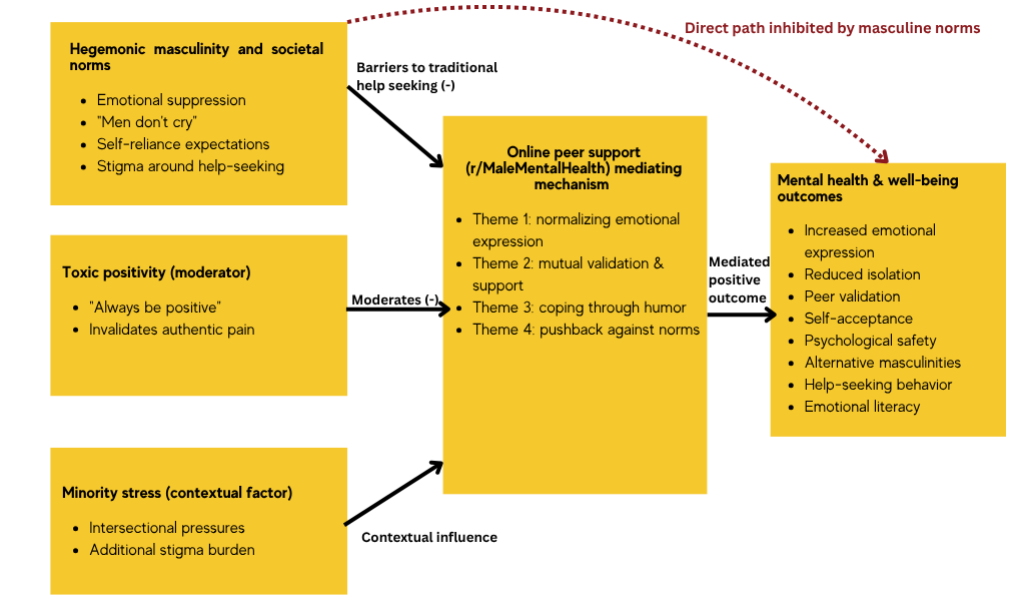
Theoretical framework: online peer support as a mediating mechanism between hegemonic masculine norms and mental health outcomes.

The theoretical framework presented in Figure 1 synthesizes findings from our thematic analysis with established theoretical perspectives to explain how men navigate mental health discourse in online peer support environments. This model positions online peer support as a critical mediating mechanism that transforms the traditionally inhibitory effects of masculine norms into positive mental health outcomes.

### Framework Components and Relationships

This framework conceptualizes online peer support, such as Reddit’s r/MaleMentalHealth and YouTube posts and comment sections, as a mediating mechanism between hegemonic masculine norms and men’s mental health outcomes. Instead of a direct, inhibited pathway from masculine socialization to poor mental health, the framework identifies a mediated route where digital peer spaces enable men to engage in emotional expression, validation, and community support.

Key mediating mechanisms include the four themes: normalizing emotional expression, mutual validation, coping through humor, and pushback against norms. These are facilitated through what we term anonymous authenticity, where men feel safe expressing vulnerability online despite societal pressures to remain stoic.

Toxic positivity is positioned as a moderator, subtly shaping how masculine norms affect participation. While it allows some emotional expression, it restricts men to “positive” displays, often silencing authentic distress. Our analysis shows how men actively resist this pressure by cocreating norms that value genuine emotional honesty over superficial optimism.

Minority stress is framed as a contextual amplifier, not merely an individual-level variable. For men facing intersectional stigma—due to race, sexuality, or other factors—online platforms often provide the only accessible support space, intensifying their relevance.

This framework contributes to theory in 3 key ways: first, it moves beyond binary masculinity models by showing that masculine norms can either inhibit or enable help-seeking depending on context; second, it reinterprets peer support theory for the digital era by replacing identity disclosure with shared emotional experience; and third, it highlights online resistance, where men collectively challenge both traditional stoicism and toxic positivity through everyday digital interactions.

Overall, the framework reflects how online communities are reshaping men’s mental health discourse, offering alternative pathways for healing, solidarity, and emotional growth. Within this framework, online peer support operates as a discursive environment that may enable norm flexibility and gradual reframing of masculine expression, rather than producing direct behavioral change. These digital interactions highlight how emotional openness is negotiated and tested within specific peer contexts, offering insight into potential pathways of masculine norm evolution.

## Methods

### Study Design

**Figure 2:**
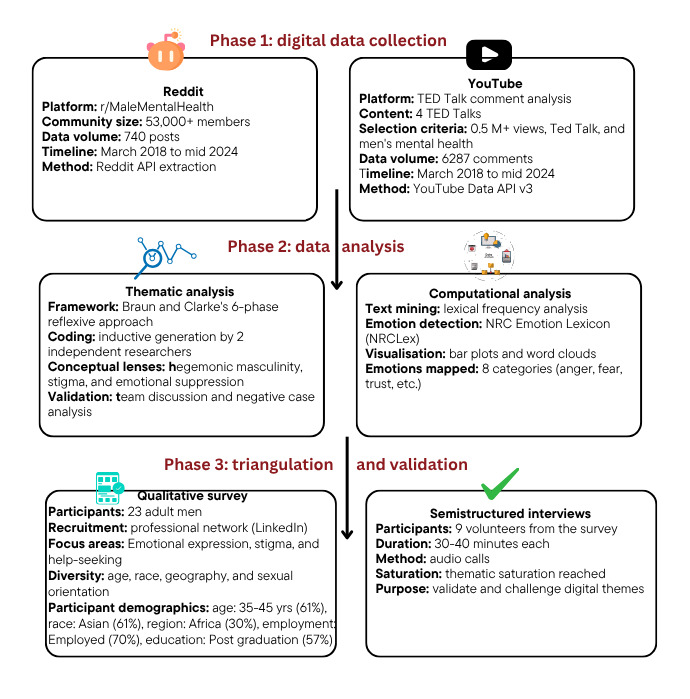
Convergent mixed methods design: data collection, parallel analysis, and triangulation phases. API: application programming interface; NRC: National Research Council.

This study used an exploratory mixed methods design using a convergent approach [51], in which qualitative and quantitative data were collected concurrently and integrated during interpretation to explore how men articulate and navigate mental health concerns in digital environments. The study size was determined by capturing all available posts from the selected Reddit thread during the collection period and all comments from the 4 YouTube videos. No additional sampling or truncation was applied. Primary data were drawn from 2 publicly accessible online platforms (Reddit and YouTube) to identify key themes and patterns in user-generated discourse. These themes were then triangulated and validated through a follow-up qualitative survey and semistructured interviews with men from diverse backgrounds (refer to Figure 2). The design was informed by reflexive thematic analysis, supported by computational text mining and emotion analysis.

### Data Sources and Sampling

#### Reddit

A total of 740 posts, including original submissions and top-voted posts, were collected from the subreddit r/MaleMentalHealth*,* a community with over 53,000 members at the time of data collection. The posts spanned from the subreddit’s creation in March 2018 through mid-2024. Data were collected using the Reddit application programming interface and included only publicly available, anonymized content. No personally identifiable information was recorded.

#### YouTube

We also extracted user comments from 4 TEDx Talks on YouTube that focused on men’s mental health. Videos were selected using purposive sampling based on three criteria: (1) central relevance to men’s mental health, (2) TEDx branding, and (3) a minimum of half a million views. Using approved access via the YouTube Researcher Program and the YouTube Data application programming interface (version 3), we extracted 6287 comments totaling over 343,060 likes in aggregate.

The selected videos were Why Big Boys Don’t Cry by Gareth Griffith (TEDxUniversityofBristol), Mental Health Crisis in Men by Allan Kehler (TEDxDalhousieU), Suffering in Silence: The Emotional Abuse of Men by Dr Timothy Golden (TEDxWallaWallaUniversity), and We Need to Talk About Male Suicide by Steph Slack (TEDxFolkestone). These videos collectively reflect diverse perspectives on male mental and emotional health, making them highly relevant for analyzing public engagement and discourse in the comment sections.

### Data Analysis

#### Qualitative Data Analysis

Primary outcomes included themes reflecting emotional expression, peer support, and attitudes toward masculinity. Secondary analytic focus examined discourse patterns and emotion lexicon categories (eg, anger, joy, and sadness) as indicators of affective tone. We used Braun and Clarke’s [52,53] 6-phase reflexive thematic analysis framework to examine patterns in Reddit and YouTube discussions on men’s mental health. The process began with data familiarization, where the primary researcher read and reread Reddit posts from r/MaleMentalHealth and high-engagement YouTube comments. Posts and comments were prioritized based on the number of likes to foreground socially endorsed discourse. In the initial coding phase, the first researcher applied open, inductive coding using Python libraries such as pandas, Natural Language Toolkit, and word cloud, allowing themes to emerge directly from the data rather than from pre-existing frameworks. These codes were then discussed with a second researcher in a process of reflexive dialogue to deepen interpretation and challenge assumptions. The goal was not to test coding consistency but to enhance analytical rigor and reflexive awareness. Collaborative discussions helped refine theme meanings and ensure sensitivity to multiple perspectives.

Codes were next grouped into preliminary themes, representing shared latent meanings within the data. In phase 4, both researchers reviewed and refined these candidate themes to ensure they were internally consistent, distinct, and sufficiently represented the dataset. Phase 5 involved clearly naming and defining the final themes, supported by the development of a thematic map to visualize intertheme relationships. Analytical credibility was strengthened through collaborative reflexivity, negative case analysis, and a shared interpretive process between researchers, ensuring that the final 4 themes captured both the emotional depth and social nuance of men’s mental health discourse across platforms. As part of reflexive rigor, researchers actively sought negative cases, instances that challenged a candidate theme, and used them to refine theme boundaries and labels. Researchers monitored thematic saturation throughout the iterative coding process, considering it reached when no substantively new codes or insights emerged during the final rounds of analysis and when subsequent data confirmed the stability of the existing thematic structure.

#### Quantitative Data Analysis

To support and deepen qualitative interpretation, we conducted lexical frequency analysis and emotion detection using the National Research Council Emotion Lexicon via the National Research Council Lexicon Python library. The most frequently occurring words were visualized using bar plots and word clouds. Emotional tone was mapped, providing a computational snapshot of the affective landscape of digital discourse. These outputs were descriptive context for qualitative interpretation and were not used as inferential evidence.

#### Mixed Methods Integration

To complement the social media findings and assess whether digital themes aligned with lived experiences, the study used a sequential triangulation approach, comprising an online qualitative survey followed by in-depth interviews. A total of 23 adult men were recruited via professional networks such as LinkedIn to complete the survey, which included open-ended questions on emotional expression, stigma, coping strategies, and help-seeking behavior. Recruitment followed a purposive and convenience sampling approach through professional networks and online well-being groups. Interviewees were selected from survey respondents who consented to follow-up. This initial phase enabled a broader capture of individual reflections and language around mental health, with several posts and comments reinforcing or extending themes identified from Reddit and YouTube.

From this group, 9 participants volunteered for follow-up semistructured interviews, which were conducted via audio calls. Each interview lasted approximately 30-40 minutes and focused explicitly on validating or challenging the themes that emerged from the online data. Interview questions were framed to test the resonance of digital narratives, such as humor, emotional scarcity, peer validation, and pushback against toxic positivity, in real-life contexts.

Survey and interview participants were primarily recruited through LinkedIn posts shared by the first author. As a result, the participant pool largely comprised professionals with higher education backgrounds and access to online networks. While this recruitment approach facilitated voluntary participation and ensured ethical transparency, it likely introduced an occupational and educational bias, which should be considered when interpreting the findings.

Importantly, thematic saturation was reached by the 7th interview, with no substantially new codes or themes emerging thereafter. This aligns with Braun and Clarke’s [54] large-scale saturation study, which found that 94% of frequent themes emerge within the first 6 interviews and 97% by the twelfth, supporting the adequacy of smaller, focused qualitative samples. Additionally, Sim et al [55] recommend a sample size of 6-15 participants for in-depth qualitative studies. Given that the primary aim was to test pre-existing digital themes rather than generate entirely new ones, the sample of 9 interviews was both methodologically sufficient and theoretically sound.

In summary, the survey and interviews provided meaningful triangulation of online discourse, offering depth, confirmation, and nuance to the digital findings, while remaining within accepted qualitative research standards for sample adequacy and saturation (Table 1).

**Table 1:** Demographic characteristics of survey participants (N=23).

Variable and category			Value, n (%)
**Age group (years)**			
	35-45	14 (60.9)	
	45-55	4 (17.4)	
	25-35	4 (17.4)	
	18-25	1 (4.3)	
**Race**			
	Asian	14 (60.9)	
	White	4 (17.4)	
	Other	3 (13)	
	Mixed race	1 (4.3)	
	Black African	1 (4.3)	
**Region**			
	Africa	7 (30.4)	
	Asia	7 (30.4)	
	Europe	4 (17.4)	
	Other	2 (8.7)	
	Australia	1 (4.3)	
	North America	1 (4.3)	
	South America	1 (4.3)	
**Employment status**			
	Employed	16 (69.6)	
	Other (student, self-employed, etc)	4 (17.4)	
	Unemployed	3 (13)	
**Education**			
	Postgraduate	13 (56.5)	
	Graduate	10 (43.5)	

### Missing Data Handling

No missing data were present in the Reddit (n=740) or YouTube (n=6287) datasets, as all publicly accessible posts and comments were extracted by the software. For the survey component, all 23 respondents completed all mandatory fields, resulting in 0% missing data. Optional open-ended responses had a 100% completion rate. Interview data were complete for all 9 participants, with no session interruptions or incomplete transcripts. Therefore, no imputation procedures or missing data analyses were necessary.

### Researcher Positionality and Reflexivity

The research team comprised a male primary researcher (first author) with 14 years of human resource management leadership experience and mental health expertise, and a female secondary researcher (second author) with an industrial psychology specialization. This gender-balanced approach provided complementary perspectives while mitigating analytical bias in examining masculine discourse dynamics. The primary researcher’s positioning as male enabled insider understanding of masculine socialization experiences, while the secondary researcher provided external validation of interpretive processes. Researchers maintained reflexive memos documenting analytical decisions and potential biases throughout data collection and analysis.

### Ethical Considerations

This study was approved by the University of Johannesburg Department of Industrial Psychology and People Management Research Ethics Committee (clearance no IPPM-2022-618[D]). All procedures were conducted in accordance with local legislation and institutional requirements. Ethical protocols were followed rigorously across all phases of data collection and analysis. Survey procedures adhered to the CHERRIES (Checklist for Reporting Results of Internet E-Surveys) guidelines [56], with a detailed checklist provided in Multimedia Appendix 1. This study also analyzed publicly available Reddit comments, where users post with an expectation of broad visibility [57,58]. As the research focused solely on analyzing public text-based discourse (without directly engaging human participants), it was exempt from formal ethical review, consistent with current regulatory interpretations [59,60]. However, following the principles of situated ethics, care was taken to protect participants from harm [26,61,62]. To minimize risks of reidentification, all usernames and personal identifiers were excluded, and findings are reported in aggregate form [58,63,64]. For the survey and interview components, all participants (N=23) provided their written informed consent via tick-box acknowledgment before accessing the questionnaire. The consent form detailed the study purpose, voluntary participation, anonymity protections, estimated time commitment, and the researcher’s contact information. Participants were informed that they could withdraw at any time without penalty. All data collection and storage adhered to privacy-by-design principles. For survey and interview participants, no personally identifiable information (names, email addresses, IP addresses, geographic locations, or other identifiers) was collected at any stage. Survey responses were automatically anonymized through Google Forms’ privacy settings, with restricted access limited to the research team. Interview notes were deidentified immediately upon completion, with no audio or video recordings made, to protect participant privacy and encourage candid discussion.

For Reddit and YouTube data, usernames and handles were systematically removed during data extraction and replaced with generic identifiers. All visual content from Reddit was recreated as schematic illustrations in Canva (Canva Pty Ltd) to prevent reverse image searching and protect user anonymity. No URLs, timestamps, or other metadata that could enable identification of original posts were retained. All data were stored on password-protected, encrypted devices, accessible only to the research team. No financial compensation, incentives, or rewards were offered to survey or interview participants. Participation was entirely voluntary and altruistic, and no employer or third-party organizations were involved in recruitment or data collection. No images in this paper contain identifiable individuals. All visual examples from Reddit were recreated as schematic illustrations in Canva, preserving thematic content while eliminating traceability to original users or posts. All narrative excerpts from online platforms were lightly paraphrased to prevent searchability while maintaining semantic meaning. This approach ensures that individual users cannot be identified despite the public nature of the original posts. Three recruitment posts were made by the first author on different days in July 2025 through his personal LinkedIn account (over 12,000 followers). The posts invited adult volunteers (aged ≥18 years) from any location to participate in an anonymous survey on men’s mental health experiences. There was no paid promotion or employer involvement. Interested individuals accessed an information sheet and provided tick-box consent before proceeding to the survey. The survey form included the following statement:

“This survey is completely anonymous—we do not ask for your name, phone number, email, or any personally identifying information (such as social security or ID numbers). As a result, no one will be able to identify you from your responses, especially within a large sample.”

At the end of the form, participants were invited to contact the researchers if they were willing to take part in a short follow-up interview about the topic. In total, 23 participants completed the survey, and 9 of them subsequently volunteered for interviews. To ensure participants felt comfortable speaking openly, no audio or video recordings were made. Instead, researchers took detailed written notes during each interview. At the start of every discussion, participants were reminded that no personal or identifying data would be shared with anyone and that their participation was voluntary.

## Results

### Overview

This section presents 4 key themes that emerged from the thematic analysis of Reddit posts, YouTube comments, and triangulated insights from survey and interview data. Collectively, these themes reveal how men use anonymous online spaces to express vulnerability, seek validation, cope through humor, and push back against societal norms of toxic positivity and stoicism. These digital communities offer men not only emotional refuge, but also opportunities to cocreate new narratives of masculinity grounded in honesty, empathy, and mutual support.

Before presenting thematic findings, we provide illustrative examples of the visual and narrative content that characterizes men’s mental health discussions on Reddit and YouTube. These examples contextualize the tone, emotional depth, and cultural critique present in the analyzed discourse.

**Figure 3:**
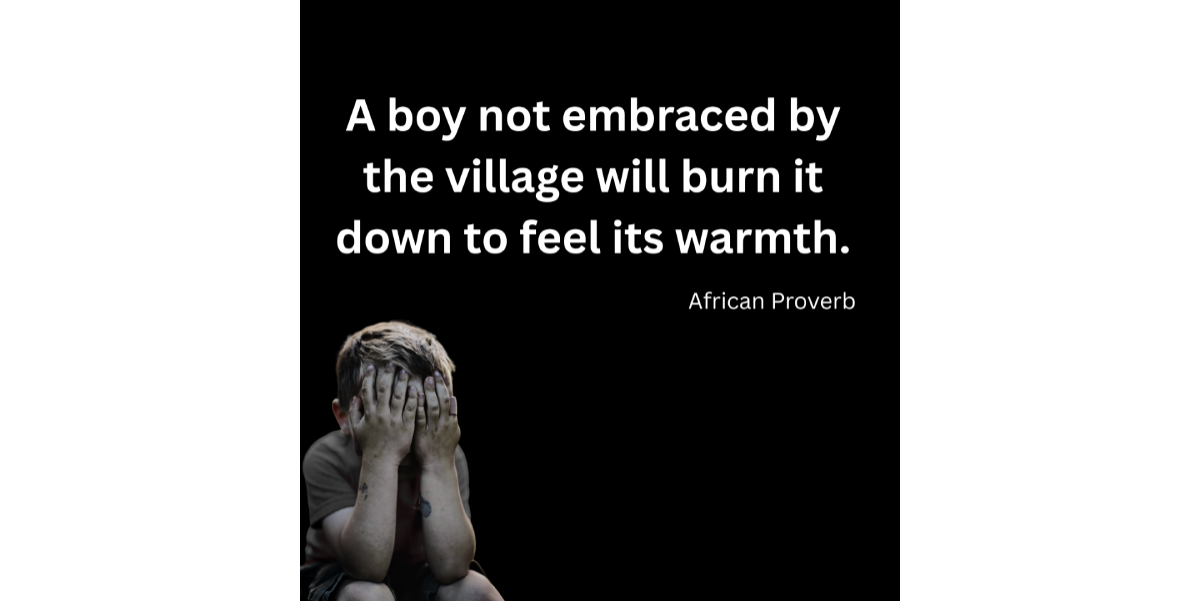
African proverb on male emotional suppression posted to r/MaleMentalHealth. Image recreated to protect user anonymity.

Figure 3 presents a widely shared African proverb addressing emotional neglect and its consequences, reflecting broader discussions about societal invalidation of men’s emotional needs. Such culturally resonant imagery frequently generated extended discussion threads exploring men’s experiences of being unseen or unsupported.

**Figure 4:**
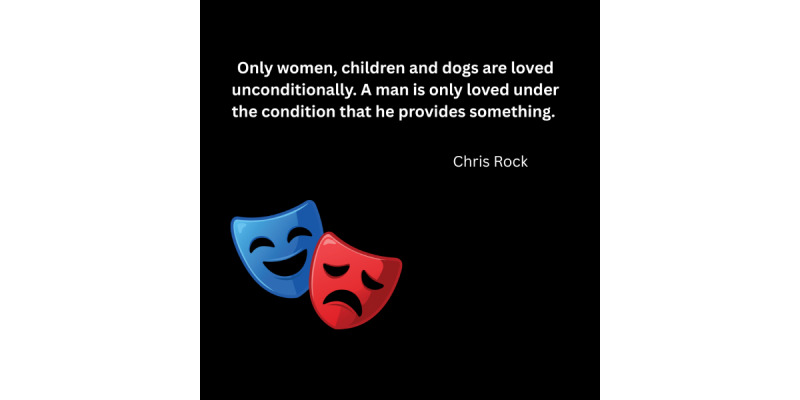
A quote attributed to comedian Chris Rock expressing cultural perception of conditional male value, widely circulated in online mental health discussions. Image recreated by authors.

Figure 4 illustrates a recurring narrative about conditional male value, captured in a quote widely attributed to comedian Chris Rock. This perception, that men are valued only for what they provide rather than for their inherent worth, appeared repeatedly across platforms and aligned with survey participants’ descriptions of feeling instrumentalized.

**Figure 5:**
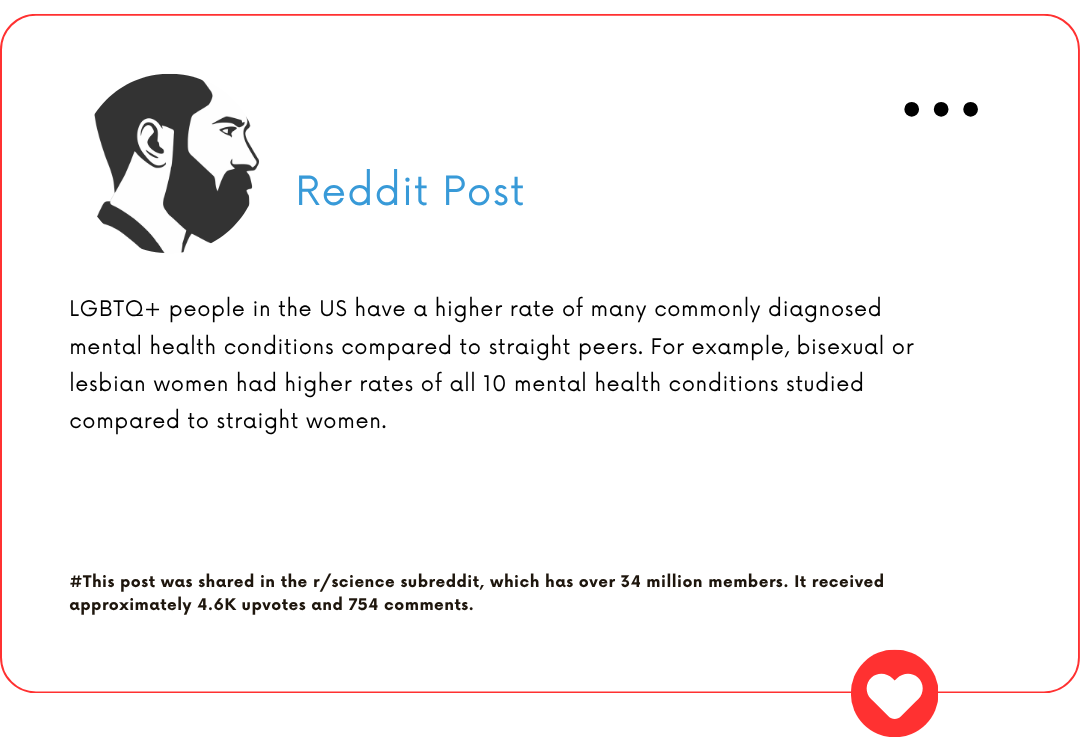
Reddit post from r/Science discussing mental health disparities among individuals who identify as Lesbian, Gay, Bisexual, Transgender, Queer or Questioning, and more (LGBTQ+). Image recreated to protect user anonymity.

Figure 5 demonstrates how online communities engage with empirical research on intersectional mental health issues, specifically LGBTQ+ mental health disparities. Posts sharing research findings often sparked discussions connecting minority stress theory to men’s experiences of multiple marginalized identities.

**Figure 6:**
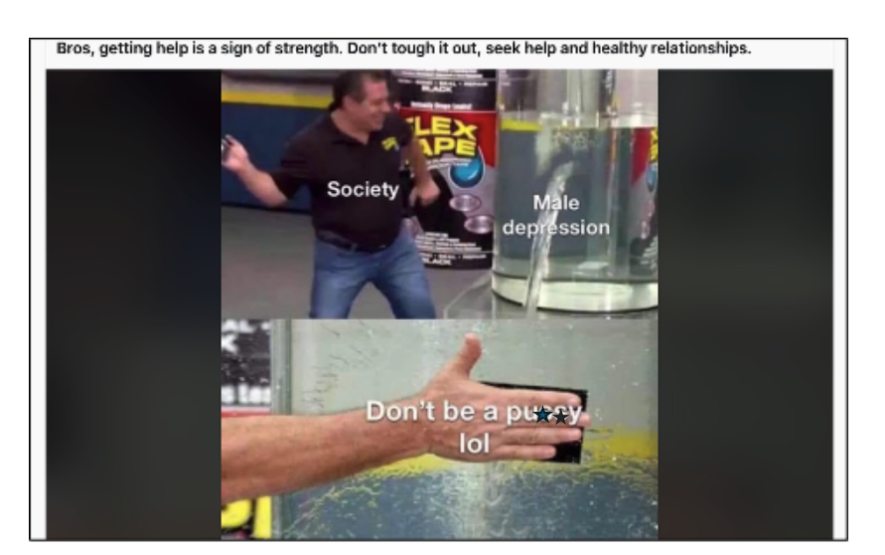
A meme posted to r/MaleMentalHealth contrasts help-seeking messaging with social mockery of male vulnerability. Image recreated by authors.

**Figure 7:**
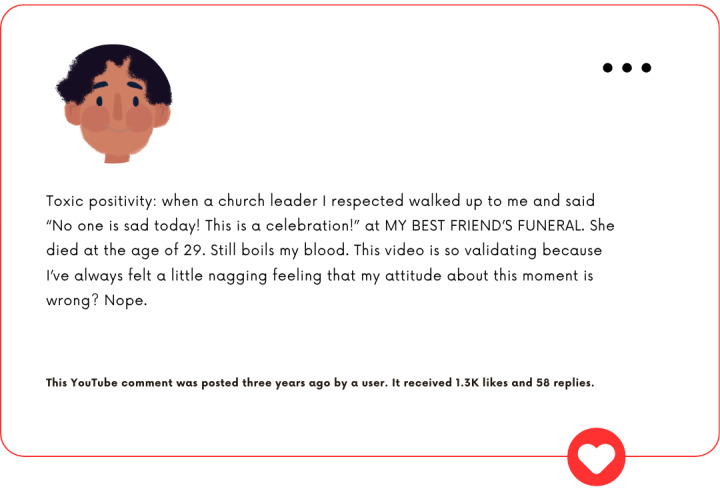
YouTube comment critiquing toxic positivity through an account of emotional invalidation at a funeral. Image recreated by authors.

Figures 6-7 exemplify the visual satire and personal testimony used to critique toxic positivity and mixed societal messages about male vulnerability. These formats allowed users to simultaneously express pain and critique cultural contradictions experienced when attempting emotional openness.

These representative examples illustrate the multimodal nature of digital mental health discourse, combining text, images, humor, and testimony to create spaces for emotional expression and social critique. The following sections present the 4 core themes that emerged from systematic analysis of this discourse.

As illustrated in Figure 2, the 4 themes were not derived from isolated data sources but through a process of systematic triangulation. Thematic patterns identified in Reddit and YouTube datasets were compared with survey and interview findings during Phase 3 of the study. This convergent approach enabled cross-validation across qualitative and quantitative strands: online expressions of emotion, humor, and peer support were examined for alignment or contrast with participants’ self-reported experiences. Convergent patterns strengthened the credibility of themes, while divergent or contradictory accounts were treated as negative cases and discussed reflexively to refine thematic boundaries. Through this process, the analysis remained grounded across multiple data sources, ensuring that interpretations reflected a holistic view of men’s mental-health discourse in digital environments.

### Theme 1: Normalizing Emotional Expression (Emotional Safety)

Participants across platforms are reclaiming emotional spaces where men can express vulnerability without judgment.

One of the most striking cross-platform insights, visible across Reddit, YouTube, and participant interviews, was the urgent need to normalize emotional expression among men. Digital spaces emerged as crucial venues where men could articulate their emotional struggles, often for the first time. These environments provided rare sanctuaries of psychological safety, where masculinity was not policed but redefined.

This theme stands in sharp contrast to hegemonic masculinity norms that discourage emotional vulnerability [17]. In many posts and comments, men directly challenged phrases such as “man up” or “be strong,” revealing how such cultural expectations had silenced them throughout their lives.

YouTube comments further echo the emotional double bind men experience when navigating vulnerability. Many users highlighted the contradictory social expectations placed on men: “When a man opens up about his emotions, they say he should man up. When a man doesn’t open up, they say it’s ‘toxic masculinity.’” This underscores the impossibility of conforming to prevailing norms without incurring judgment. Others reflected on direct personal invalidation, such as one man who shared, *“*I have a female friend and she called me weak for having depression and opening up my anxious thoughts.” These narratives illustrate how emotional suppression becomes a survival strategy rather than a preference. As one commenter put it, “I only cry in private. Many women don’t like sensitive men and see it as a sign of weakness.” Another added with bitter irony, “Do you know who isn’t allowed to talk about male suicide? Spoiler… Males.” These voices reinforce the need for anonymous digital platforms where emotional honesty is possible, spaces where men can finally speak without fear of ridicule or erasure.

**Figure 8:**
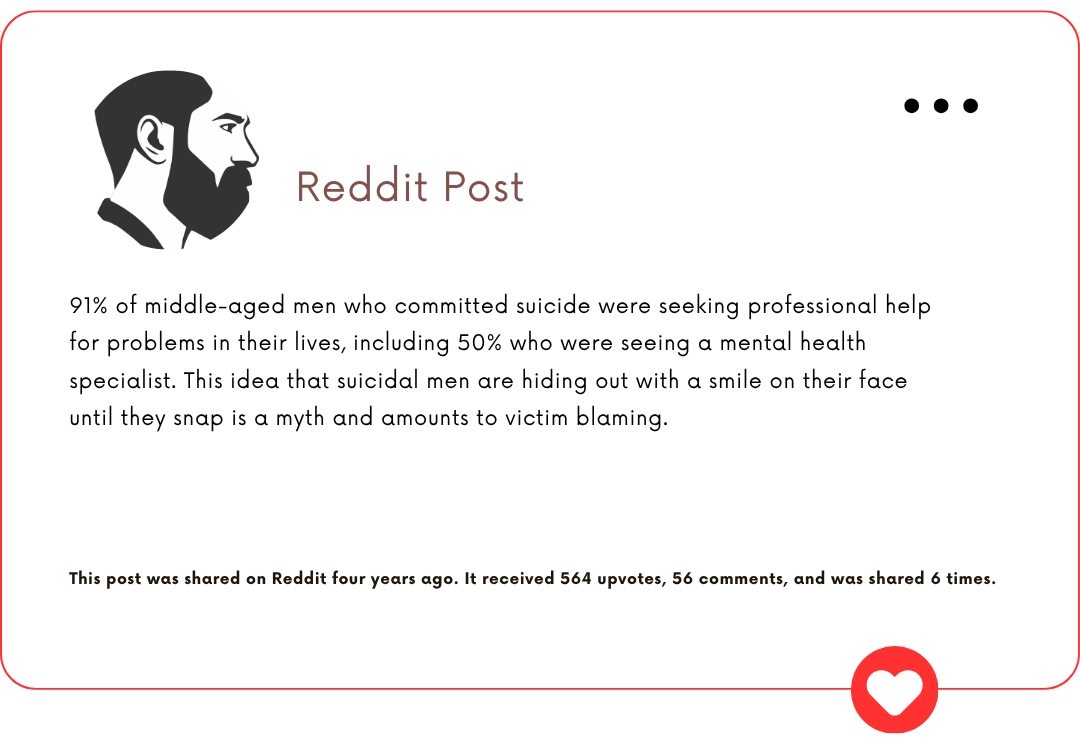
Reddit post reframing male suicide as systemic neglect rather than individual silence. Image recreated by authors.

Another Reddit post (Figure 8) disrupted the popular myth that suicidal men show no visible signs before taking their lives. The user cited a study showing that 91% of middle-aged men who died by suicide had already sought professional help, arguing that cultural norms often ignore or downplay visible distress in men. They reframed this silence as a form of societal neglect, not individual failure.

The emotional impact of such neglect was vividly captured in Multimedia Appendix 2, where a user described a partner’s reaction to basic physical comfort: “My boyfriend nearly cried when I stroked his head. He told me no one ever did that for him. It broke my heart that something so simple could mean so much.”

This idea, that even small gestures can hold emotional weight, was echoed in survey responses, where 20 of 23 men explicitly reported that they do not feel society provides a safe space to discuss mental health. This lack of openness was not abstract; it shaped lived experiences across age, profession, and background.

In one interview, an Indian senior manager in Human Resources recounted: “I didn’t even know I was having a nervous breakdown. No one taught me in school, college, or even at work, and I work in HR, for God’s sake! Thank God a friend insisted I see a doctor. You should’ve seen my face when the doctor told me it was a nervous breakdown. I was shell-shocked. I did not have anyone to share this with!”

YouTube comments reinforced this longing for validation. One widely liked post (6000+ likes) stated: “It’s okay to feel strong negative emotions. It’s okay to talk about it. This is the first time I don’t feel broken. Reading these comments gives me hope.”

Several men in the survey expressed a yearning for simple acts of care, such as a hug, a listening ear, or someone to validate their feelings, but noted that such support was rarely available to them. This emotional void was so pronounced that some respondents described turning to technology for comfort. One North American participant, a graduate-level man, remarked: “AI that validates your opinions and uses Socratic questioning to guide you to recovery.” His comment highlights a growing trend: in the absence of safe interpersonal spaces, some men are turning to artificial intelligence tools in search of empathy, reflection, and emotional guidance. This underscores the urgency of creating more human-centered environments in which emotional expression is not only permitted but also actively nurtured.

Collectively, these sources demonstrate that men are investing in anonymous digital spaces, not for distraction but for connection and self-understanding. Platforms such as Reddit and YouTube are not merely hosting content; they are actively reshaping emotional norms through peer-to-peer empathy. What emerges is a collective rewriting of masculinity, one that prioritizes emotional literacy, mutual support, and the simple radical act of being heard.

### Theoretical Interpretation

Theme 1 supports critiques of hegemonic masculinity, which frames emotional openness as weakness [21]. Platforms such as Reddit and YouTube serve as “digitally mediated sanctuaries,” enabling men to express vulnerability without fear of judgment. These anonymous spaces challenge dominant norms by normalizing emotional need and fostering peer validation, echoing calls for healthier, psychologically supportive masculinities [35].

### Theme 2: Mutual Validation and Peer Support (Belonging and Affirmation)

Anonymous online platforms serve as emotionally validating spaces where men’s struggles are met with empathy, solidarity, and rare affirmations—often absent in offline life.

Anonymous digital communities such as Reddit and YouTube are emerging as vital ecosystems of emotional support, where men’s disclosures are met with empathy, affirmation, and solidarity, which is often missing in their offline lives. In contrast to traditional environments such as workplaces, families, or male peer groups, where vulnerability may be mocked or dismissed, these online platforms provide men with a rare sense of being heard and validated.

Across Reddit threads, users frequently recounted how even the smallest compliments, often decades old, left lasting emotional imprints. One user wrote, “A girl telling me I have a nice smile when I was 14 still stands out as one of the nicest compliments I’ve ever received. I’m now 24.” Another recalled, “I got a compliment from a coworker in 2010 about how I smelled really nice… I still buy the same deodorant and bath soap to this day and remember her compliment every time I use them.” These memories reveal not only emotional scarcity but also the hunger for non–performance-based appreciation, a form of validation rarely offered to men in face-to-face settings.

This emotional deprivation was echoed in our primary data. A 28-year-old South African respondent shared in the survey, “I can’t remember the last time anyone said something kind to me that wasn’t about my work.” Others spoke of longing for connection and affirmation that never materialized in their immediate circles. Of the 22 men surveyed, a majority answered “No” when asked if they had a safe space in society to talk about mental health. For many, online communities have become surrogate support systems, especially in the absence of affirming offline relationships.

Comments from the YouTube section of male mental health videos mirrored these concerns. One viewer reflected, “Recently, I told my mom how depressed I was every day. The first thing she asked was if I was still able to do my job properly… then she switched the subject to herself… not once acknowledging my depression.” Another added, “I love how women are the ones advocating for men’s rights, depression, suicide… because if men were doing it, we’d be labelled as sexist or misogynistic.”

An employed African interviewee, a graduate, gave a poignant account of grief and peer dismissal: “Forget about peer validation and support from other men! The amount of toxic advice men get is unbelievable. In my circles, it’s considered embarrassing if a young man is crying at the funeral of a loved one, rather than comforting the young one at his loss.” This sense of isolation, minimization, and toxic resilience underscores why digital communities are so significant. They offer the validation that offline contexts frequently deny.

These dynamics are not only expressed in words but also visually represented through memes that resonate widely within these communities. As illustrated in Multimedia Appendix 3, one viral Reddit post noted: “The average guy is so starved for positive attention that a simple compliment is enough to get him interested in you.” The meme attracted thousands of upvotes, reflecting its broad emotional resonance among men online.

In this environment, digital peer support becomes a mechanism of emotional reclamation. Common affirmations such as “We should complement each other more often” or “Everyone’s life is precious” reflect a growing counter-narrative that values emotional expression, mutual care, and nontransactional affirmation. Rather than perform masculinity through stoicism or silence, men in these communities are rewriting the script—collectively shaping new norms grounded in emotional openness.

As one participant explained, reflecting on the significance of online peer environments and artificial intelligence–based support systems: “What I get from social media is not therapy, but perspective. People here remind me that it’s okay to feel, and that I’m not broken for wanting to be seen.”

### Interpretation in Theoretical Context

Theme 2 reflects challenges to hegemonic masculinity, which discourages emotional openness and mutual validation [21]. Reddit and YouTube function as “safe digital intimacy zones” where men exchange empathy and emotional support. This aligns with Sense of Community Theory [65], as these online spaces foster belonging, shared emotional connection, and nonstigmatizing expressions of vulnerability.

A small subset of posts framed humor as a form of avoidance rather than connection. These were treated as negative cases and used to refine the thematic boundaries. Accordingly, in Theme 3, we highlight how humor operates along a continuum, from emotional deflection to genuine bonding, depending on context and intent.

### Theme 3: Coping Through Humor and Irony (Emotional Deflection and Connection)

Humor, irony, and self-deprecation emerge as culturally sanctioned tools that allow men to express psychological pain while preserving masculine norms of emotional restraint.

Across Reddit and YouTube, men often ridicule societal expectations such as “man up” and “don’t be a pu**y,” not to diminish their pain, but to reframe it in socially palatable ways. For instance, one Reddit user sarcastically asked, “Can we change ‘Be a man’ to mean it’s okay to cry?” while another remarked, “I cried myself to sleep after a depression battle but refused to tell my girlfriend because I’d look weak.” Such posts illuminate the tension between inner emotional needs and external masculine scripts. This emotional double-bind is often mediated through visual satire and dark memes, an aesthetic of shared struggle disguised as humor.

YouTube comments similarly reflect this mode of coping. One user noted, “Dead people receive way more flowers than the living because regret is stronger than gratitude,” while another quipped, “If anything happens to a guy, it’s just considered funny.” These sharp observations lay bare the emotional neglect men experience, and the ironic laughter used to endure it.

This sentiment is reflected in interviews and survey responses as well. A South African participant stated, “I usually joke about my stress before I talk about it seriously… it makes it less awkward.” Another shared, “Laughing at it feels safer than admitting how close I am to burning out.” Humor, then, becomes a socially acceptable entry point for serious conversations that might otherwise be dismissed. A participant added in the interview: “It has been the best skill I have acquired. It helps me navigate social awkwardness and build relationships. It keeps me from shutting down.” Others echoed this pattern of “laughing through the pain” in emotionally invalidating environments.

### Visual Satire as Collective Commentary

This use of humor is also poignantly captured in popular memes. One widely upvoted post (Multimedia Appendix 4) shows a drowning figure labelled “Men’s mental health” reaching for help, only to be slapped away by a hand labelled “Society” shouting “Be a man”. The meme’s caption simply reads: “Yikes. Accurate though.”

Another meme (Multimedia Appendix 5) uses cartoon imagery to depict a man loading a revolver marked “Men having mental health issues” with bullets labelled “Be a man,” a chilling metaphor of how societal messaging weaponizes masculinity against men themselves.

### Interpretation in Theoretical Context

Theme 3 illustrates how humor acts as a culturally acceptable outlet for emotional pain under hegemonic masculinity, where vulnerability is often ridiculed [21]. In online spaces, humor becomes a tool of resistance, allowing men to share distress while preserving social acceptability. This aligns with the sense of community theory [65], as shared laughter fosters connection, emotional safety, and subtle defiance of rigid masculine norms.

### Theme 4: Pushback Against Toxic Positivity and Societal Norms (Emotional Resistance)

This theme reflects how men are actively rejecting societal pressures that dismiss emotional pain and enforce performative strength, advocating instead for a more honest, compassionate masculinity.

This theme captures men’s rejection of societal scripts that silence emotional expression and promote performative strength. Across platforms, participants challenge cultural expectations such as “man up” or “stay strong,” which invalidate their distress and leave little room for vulnerability. In doing so, they advocate for a redefinition of masculinity rooted in emotional honesty.

A widely upvoted YouTube comment criticized the double standards faced by men attempting to open up: “Women: ‘Men should talk about their feelings. It’s okay to feel vulnerable.’ Also, women: Sensitive men are needy and weak. I only date real men.’”

On Reddit, sarcastic memes and personal testimonies echoed this dissonance. One user posted an image highlighting that “Will Smith crying turned into a meme is proof that the mental health of men is not taken seriously” (Multimedia Appendix 6), while another shared a visual list reminding readers that “boys’ feelings are valid too” (Multimedia Appendix 7). These posts reflect a broader cultural dismissal of male distress, often amplified by humor or invisibilization.

This rejection of emotional suppression was also voiced in interviews and open-ended survey responses. One South African respondent shared: “Yes. I now have a much more difficult time crying, even when alone, and I was forced to close off from my family, which resulted in me being kicked out of my home at 18.” Another stated, “Therapy can’t look at you when you’re failing… men are never taught how to speak about their experiences in ways others can understand. It’s a skill that has to be learned.” Several participants noted the internal conflict caused by having to appear strong while feeling shattered inside.

One Reddit post humorously critiqued performative masculinity: “I hate how, as a man, the solution to life’s problems is inevitably ‘lift weights.’ Academic stress? Lift. Marital issues? Lift. Depression? Lift. At this point, it wouldn’t surprise me if someone said lifting cures cancer.” These ironic retorts offer a subversive form of resistance against toxic positivity.

Despite this pushback, the desire for compassion was clear. A simple yet powerful Reddit post read: “I don’t want physical intimacy. I just want someone to hold me.” Others shared artwork and messages such as “Men need to know it’s okay to cry, to be vulnerable, to seek support” and “Your body is beautiful bro” (Multimedia Appendix 8), reinforcing the call for emotional affirmation rather than judgment or dismissal.

The cumulative effect of these expressions—memes, confessions, and affirmations—illustrates a growing resistance to outdated norms of stoicism. As captured in a Reddit quote, “Men are told to open up, and then to shut up. No wonder they don’t feel safe talking.” (Multimedia Appendix 9). These narratives point to a desire not only to speak but also to be heard, seen, and validated as emotionally complex human beings.

### Interpretation in Theoretical Context

Theme 4 highlights resistance to hegemonic masculinity, where stoicism and strength are prioritized at the expense of emotional well-being [17]. By challenging tropes such as “man up,” men expose the psychological cost of these norms. This resistance also aligns with inclusive masculinities theory [66], as men begin to legitimize emotional openness and redefine masculinity in more diverse and supportive ways.

### Linguistic and Emotional Patterns in Online Discourse on Men’s Mental Health

To complement the thematic findings, Python-based linguistic and emotion analyses were conducted on the Reddit and YouTube datasets. The word frequency analysis (refer to Multimedia Appendix 10) showed that frequently used terms—such as men, women, suicide, depression, and society—center discussions on gender, mental health struggles, and societal expectations.

The emotion analysis using the National Research Council Emotion lexicon (refer to Multimedia Appendix 11) revealed a mix of positive and negative emotions, with prominent expressions of sadness, fear, trust, and anger. This pattern suggests that while users often voice pain and vulnerability, the discourse also contains resilience, empathy, and occasional hope.

Together, these lexical and emotional trends underscore that online platforms function as spaces where men articulate distress and co-construct supportive narratives around mental health.

## Discussion

### Principal Findings

Four interrelated themes emerged: (1) normalizing emotional expression, (2) mutual validation and peer support, (3) coping through humor and irony, and (4) pushback against toxic positivity. Anonymous platforms functioned as “digitally mediated sanctuaries,” enabling emotional disclosure with reduced social risk.

First, anonymous platforms such as Reddit and YouTube provided “safe digital intimacy zones” where men could express vulnerability without fear of judgment. This finding supports the notion of “anonymous authenticity,” where the protective veil of anonymity fosters genuine emotional disclosure and enables men to renegotiate masculinity as open rather than stoic.

Second, mutual validation and peer support emerged as critical. Men consistently offered one another affirmation and understanding, which are emotional resources often unavailable offline. Online forums became surrogate support systems, echoing Sense of Community Theory [65], which emphasizes belonging, emotional connection, and fulfillment of needs. These peer-led spaces allowed men to construct alternative masculine identities grounded in openness and care.

Third, humor and irony served as culturally acceptable coping mechanisms. Men used dark jokes and memes to express distress while maintaining social cohesion. Rather than trivializing their experiences, shared humor acted as a collective pressure valve and reinforced emotional bonds within the community.

Fourth, participants explicitly pushed back against toxic positivity and rigid societal norms. They rejected platitudes such as “man up” or “just lift weights,” critiquing the invalidation of men’s emotional pain. These narratives marked a grassroots resistance to hegemonic masculinity through satire, storytelling, and emotional honesty.

Overall, our findings suggest that online communities enable men to reclaim their emotional voice and mitigate masculine stigma. These platforms serve both as safe spaces and informal “emotional classrooms,” where men can develop relational skills, resist stigma, and foster more inclusive models of masculinity, transforming vulnerability into strength and connection.

### Comparison With Prior Work

Our findings align with established literature on masculinity and mental health stigma, confirming the persistent influence of hegemonic norms that equate vulnerability with weakness [7,17]. Consistent with prior studies, participants described delaying help-seeking due to pressures to appear emotionally invulnerable [8,9]. This emotional suppression often led to shame, isolation, and maladaptive coping, reproducing a cycle noted in earlier research.

However, our study expands on this understanding by highlighting how digital peer spaces actively disrupt this cycle. While prior work has acknowledged men’s preference for informal support [1], our analysis reveals how online forums, such as r/MaleMentalHealth posts and TEDx comment sections, serve as therapeutic spaces. These communities enable candid expression and validation, suggesting that masculine norms can be moderated by context. Wagner and Reifegerste [19], our observational data indicate men thriving in environments that legitimize emotional openness.

We also extend existing work on emotional deprivation. Past research has noted that men receive less social support [67], but our participants’ deep emotional impact from small compliments reveals how acute that scarcity is. Similarly, our findings on humor build on Samson et al [68], showing how humor online acts not as avoidance but as a shared coping mechanism, fostering solidarity and facilitating deeper conversations.

Our theme on toxic positivity adds a gendered nuance to emerging critiques of this phenomenon [12,13]. Men in our study viewed forced cheerfulness as an extension of masculine pressure, invalidating pain under the guise of optimism. Unlike earlier pessimistic views, however, our data show men resisting this dynamic by affirming each other’s right to struggle.

Theoretically, our work integrates hegemonic and inclusive masculinity perspectives [21,66], illustrating that traditional norms coexist with more emotionally expressive masculinities, especially among younger users. We also apply peer support theory [69,70] and introduce the idea of “collective authenticity,” a community-based norm of truth-telling and support forged in anonymous online environments.

In summary, our study both confirms and extends prior scholarship. It demonstrates that while men remain constrained by cultural expectations, they are also influencing masculinity from the ground up through online peer support. This shift suggests future interventions may be more effective when they amplify organic, community-led approaches rather than solely relying on institutional efforts.

While the present findings highlight how online peer spaces can facilitate emotional openness and mutual validation, they should be interpreted as evidence of discursive potential rather than confirmed behavioral or cultural transformation. The study’s cross-sectional and qualitative design does not allow for causal inference or longitudinal observation of change. Moreover, the men engaging in these online communities represent a self-selecting group already predisposed toward openness and help-seeking. As such, these findings illustrate how progressive masculinity discourses are articulated and negotiated in supportive digital environments, not how widespread they are across all male populations.

While online peer spaces can serve as supportive environments that validate men’s emotional expression, they are not without risks. The same anonymity that enables openness may also facilitate misinformation, reinforce maladaptive norms, or create exclusionary dynamics that privilege certain masculine voices over others. For instance, echo-chamber effects can amplify oversimplified narratives about strength or self-reliance, while peer validation may occasionally normalize avoidance or emotional detachment. Recognizing these tensions situates our findings within a more nuanced understanding of digital peer support—one that acknowledges both its potential to foster norm flexibility and its susceptibility to reproducing aspects of hegemonic masculinity.

### Implications for Practice

This study offers several actionable insights for improving mental health support for men. Recognize the Value of Online Peer Support

Anonymous online forums offer authentic spaces for emotional expression. Clinicians and organizations should consider these as legitimate supplementary supports and may guide hesitant clients to engage with them. Collaborating with community moderators can help disseminate trustworthy resources in user-friendly ways.

#### Encourage Positive Peer Modeling

The power of mutual validation suggests a need for peer-led support spaces. Workplace- or campus-based men’s circles that promote compliments, empathy, and open sharing can counter emotional neglect [69,70]. Clinicians should also validate male clients’ emotional disclosures, modeling empathy that many men rarely receive.

#### Address Toxic Positivity Directly

Campaigns must go beyond “it’s okay to talk” and instead teach others how to respond constructively when men open up. Emotional first-aid training for teachers, coaches, and families can help shift from minimizing distress to holding space for it [71], normalizing sentiments such as “I’m here for you” over “stay strong.”

#### Use Humor as a Gateway

Humor often serves as an emotional entry point. Mental health initiatives can adopt a lighter tone, through memes, jokes, or relatable content, to build rapport before addressing deeper issues. Validating this approach makes support more accessible and engaging.

Ultimately, interventions should be co-designed with men, building on grassroots peer support already thriving online. By bridging informal digital spaces and formal care systems, practitioners can help men feel equally safe opening up in therapy as they do on Reddit.

### Strengths and Limitations

This study has several notable strengths. Chief among them is its triangulated, multimodal design, combining thousands of Reddit posts and YouTube comments with survey and 9 interview responses. This convergence allowed us to validate key themes across platforms and formats, enhancing credibility. The unusually large volume of qualitative data enabled both depth (narrative insight) and breadth (textual analysis), a rare strength in research on men’s mental health. Our use of Braun and Clarke’s [72] 6-phase thematic analysis provided methodological rigor, with themes grounded in participants’ actual language. Reflexive practices, such as team discussions to check biases, further bolstered trustworthiness.

Another strength lies in our theoretical integration. By aligning findings with frameworks such as hegemonic masculinity, online disinhibition, and Sense of Community Theory, we situate insights within broader scholarship. The study also offers strong ecological validity, as it analyzes real-world online discourse, not lab responses. The inclusion of men from varied regions and age groups enhances the cross-cultural relevance of our results.

However, some limitations must be acknowledged. Our sample, comprising self-selected commenters and volunteers, may not represent all men, especially those who remain silent or align strongly with traditional norms. The anonymity of online platforms also limits demographic certainty, and while we focused on upvoted posts and comments to reduce outlier bias, quieter dissenting views may be underrepresented. The interpretive nature of thematic analysis, while valuable for depth, introduces potential subjectivity; we addressed this through reflexive dialogue rather than intercoder reliability metrics, per Braun and Clarke’s [52] guidelines.

A key limitation of the study lies in the demographic homogeneity of the qualitative sample. The 9 interview participants and 23 survey respondents were drawn mainly from professional and higher-education backgrounds, reflecting the reach of the LinkedIn recruitment channel. This limits the transferability of the findings to broader populations of men with different socioeconomic or cultural contexts. The qualitative insights should therefore be understood as illustrative of discourse patterns within this demographic rather than generalizable to all men. Nevertheless, triangulation with a much larger corpus of Reddit and YouTube data provided valuable contextual grounding that strengthened interpretive credibility.

We also lack data on mental health outcomes, that is, whether participating in these forums leads to tangible help-seeking or symptom relief. The cross-sectional design and focus on English-language platforms further limit generalizability, especially to non-Western or nondigital populations. Finally, while our focus was on supportive dynamics, potential risks such as misinformation or unqualified peer advice in online spaces remain underexplored.

Despite these limitations, this exploratory study provides a nuanced and empirically grounded picture of how men are negotiating emotional stigma and building community in digital spaces. It lays the groundwork for future, outcome-focused research in this domain.

### Future Directions

Building on our findings, future research should explore the long-term impact of online mental health communities on men’s help-seeking and emotional well-being. Longitudinal studies can assess whether engagement in forums such as Reddit leads to sustained improvements or increased uptake of professional support.

Further work should broaden demographic and cultural scope, especially focusing on underrepresented groups such as older men, adolescents, non-English speakers, and men from LGBTQ+ or racial minority. Understanding how different identities shape digital help-seeking could improve inclusivity in support strategies.

The themes of humor, anonymity, and peer validation offer strong foundations for developing gender-sensitive interventions. Future studies could test digital peer support tools, such as apps or moderated groups, that intentionally integrate these features, measuring their impact on distress, stigma, and connection.

There is also rich potential in exploring symbolic and narrative practices. Studies could examine how acts such as posting anonymously, wearing symbols, or sharing memes promote resilience. Interventions based on storytelling or expressive creativity may offer accessible therapeutic outlets for men.

Finally, attention must be paid to the downsides of online support. Future work should explore moderation strategies, misinformation risks, and how men distinguish helpful from harmful advice in digital spaces. Strengthening these forums without undermining openness is a key challenge.

A multidisciplinary approach, blending psychology, data science, public health, and gender studies, will be crucial to understanding and optimizing how digital communities can support men’s mental health at scale.

### Conclusions

This study explored how men navigate mental health challenges through anonymous digital platforms and personal narratives. The central finding is that men are increasingly expressing emotional vulnerability in online spaces where they feel safe and heard, challenging persistent stereotypes about masculine emotional suppression. Reddit, YouTube, and similar platforms have become vital outlets for peer support, particularly for men underserved by traditional mental health pathways.

Through humor, shared struggle, and candid dialogue, men are cocreating alternative narratives of masculinity that value empathy over stoicism and connection over silence. These organic support systems, while informal, often provide the understanding and validation men struggle to find in offline contexts. The analysis demonstrates that masculinity is not a fixed barrier but a dynamic set of norms that can shift through community and conversation.

This study makes 4 key contributions to the field. First, methodologically, it combines large-scale digital discourse analysis with qualitative triangulation across multiple platforms and data sources, providing both breadth and depth rarely achieved in men’s mental health research. Second, theoretically, it extends inclusive masculinity theory into anonymous online contexts through empirical evidence, demonstrating how digital affordances enable masculine norm negotiation. Third, it introduces the concept of “digitally mediated sanctuaries” to describe online spaces where men practice vulnerability and mutual support with reduced social risk. Fourth, from an infodemiological perspective, it demonstrates how mental health information and peer support narratives circulate and gain legitimacy within male-dominated online communities, revealing patterns of information flow that differ from traditional health communication channels.

The real-world implications are significant for practice and policy. Mental health professionals can design gender-sensitive digital interventions that incorporate features men already use organically, such as humor, anonymity, and peer validation, rather than imposing clinical frameworks that may feel alienating. Public health communicators can develop campaigns that leverage the peer-to-peer dynamics and visual culture already thriving in these spaces. Organizations can recognize and support online peer networks as complementary systems that reduce help-seeking barriers and provide alternative pathways to emotional support for men hesitant to access formal services.

In essence, this research offers a hopeful message. When given safe, authentic spaces, men not only speak but also listen, validate, and transform each other. The path forward lies in amplifying these grassroots movements and designing mental health strategies that reflect how men already connect, cope, and care. Rather than viewing digital peer support as a substitute for professional care, practitioners and policymakers should understand it as a valuable entry point that meets men where they are. By bridging informal online communities with formal support systems, we can create more inclusive and effective approaches to men’s mental health that honor both the challenges men face and the creative solutions they are already building together.

## Data Availability

The data that support the findings of this study are not publicly available due to privacy and ethical restrictions but are available from the corresponding author upon reasonable request.

## Appendix

Multimedia Appendix 1 CHERRIES (Checklist for Reporting Results of Internet E-Surveys) for the men’s mental health online study.

Multimedia Appendix 2 Reddit post illustrating emotional deprivation and validation scarcity among men.

Multimedia Appendix 3 Meme from r/MaleMentalHealth depicting the impact of compliment scarcity on men.

Multimedia Appendix 4 Meme illustrating societal dismissal of men's mental health.

Multimedia Appendix 5 Cartoon depicting masculine norms as self-destructive.

Multimedia Appendix 6 Meme critiquing public mockery of male emotional expression.

Multimedia Appendix 7 Meme capturing male-specific emotional pain.

Multimedia Appendix 8 Meme depicting body shaming directed at men.

Multimedia Appendix 9 Post encapsulating contradictory social expectations around male vulnerability.

Multimedia Appendix 10 Top 30 most frequent words in Reddit posts and YouTube comments on men’s mental health.

Multimedia Appendix 11 Emotion distribution in Reddit posts and YouTube comments based on the National Research Council lexicon, showing the frequency of emotion-associated words across 8 categories plus positive and negative valence.

## Abbreviations

CHERRIES: Checklist for Reporting Results of Internet E-Surveys
LGBTQ+: Lesbian, Gay, Bisexual, Transgender, Queer or Questioning, and more
