# Supplementary material for: Online Discussions of Men’s Mental Health on Reddit and YouTube: Cross-Sectional Mixed-Methods Infodemiological Study
Source: JMIR Infodemiology. 2026 May 5;6:e81315. doi: 10.2196/81315 (PMC13187704; doi:10.2196/81315)
Supplement: Multimedia Appendix 1 [file infodemiology_v6i1e81315_app1.docx]

| **Item Category** | **Checklist Item** | **Explanation** | **This Study** |
| --- | --- | --- | --- |
| Design | Describe survey design | Describe the target population, sample frame. | An open, anonymous web-based survey was distributed via three LinkedIn posts by the first author in July 2025. Convenience sampling was used. |
| IRB and Consent | IRB approval | Mention whether the study has been approved by an IRB. | Ethical clearance obtained from the University of Johannesburg (clearance number included in paper). |
| IRB and Consent | Informed consent | Describe the informed consent process. | Tick-box consent obtained before survey start. Participants were informed about anonymity, purpose, voluntary participation, and estimated completion time. |
| IRB and Consent | Data protection | Describe mechanisms for data protection. | No personal identifiers collected. Data stored securely on an encrypted drive; survey hosted via Google Forms with restricted researcher access. |
| Development and pre-testing | Development and testing | How was the survey developed and tested? | The survey was developed by the research team to complement qualitative analyses of Reddit and YouTube data by capturing men’s self-reported experiences of mental health, emotional expression, and online help-seeking. |
| Recruitment process | Open vs closed survey | Was the survey open to all or limited (password-protected)? | Open survey accessible to anyone via LinkedIn post; no password required. |
| Recruitment process | Contact mode | How were participants contacted? | Survey link shared publicly on LinkedIn; no private invitations. |
| Recruitment process | Advertising the survey | Describe how and where the survey was advertised. | Announced via three personal LinkedIn posts by the first author (12,000+ followers). No paid promotion or employer involvement. |
| Survey administration | Web/E-mail | Type of e-survey. | Web-based Google Form survey; responses automatically captured and exported for analysis. |
| Survey administration | Context | Describe the context of the recruitment platform. | LinkedIn is a professional networking platform. Recruitment likely attracted educated and employed adults, influencing sample composition. |
| Survey administration | Mandatory/voluntary | Was the survey mandatory or voluntary? | Participation was entirely voluntary. |
| Survey administration | Incentives | Were incentives offered? | No incentives or rewards were offered. |
| Survey administration | Time/Date | Timeframe of data collection. | Survey conducted in July 2025 across three LinkedIn posts published on different days. |
| Survey administration | Randomization of items | Were items randomized? | No randomization was used; all participants received the same questionnaire. |
| Survey administration | Adaptive questioning | Was adaptive questioning used? | No adaptive questioning; all participants answered the same set of questions. |
| Survey administration | Number of items | Number of questions per page. | Single-page Google Form with approximately 14 questions. |
| Survey administration | Number of screens | Over how many pages was the questionnaire distributed? | Single-screen form. |
| Survey administration | Completeness check | Was a completeness check done? | Mandatory fields ensured completion of key items; optional non-response options included. |
| Survey administration | Review step | Could respondents review or change responses? | Respondents could review and modify answers before submission. |
| Response rates | Unique site visitor | Define how unique visitors were counted. | Google Forms does not track IPs; uniqueness is inferred from total form submissions. |
| Response rates | View rate | Ratio of unique survey visitors/unique site visitors. | Not applicable, as the link was posted on social media and no site visit data were recorded. |
| Response rates | Participation rate | Ratio of visitors who agreed to participate/first page visitors. | All who opened the form and ticked consent proceeded; participation rate estimated at 100% of form starters. |
| Response rates | Completion rate | Ratio of users who finished the survey/users who agreed to participate. | 23/23 completed surveys; completion rate 100%. |
| Preventing multiple entries | Cookies used | Were cookies used to prevent duplicates? | No cookies were used; duplicate responses unlikely due to small, voluntary sample. |
| Preventing multiple entries | IP check | Was the IP address used to prevent duplicates? | No IP tracking conducted to preserve anonymity. |
| Preventing multiple entries | Log file analysis | Were log files analyzed to detect duplicates? | No log file analysis conducted. |
| Preventing multiple entries | Registration | Was registration required? | Open anonymous access; no registration required. |
| Analysis | Handling of incomplete questionnaires | How were incomplete questionnaires handled? | All 23 surveys were complete; no partial entries were excluded. |
| Analysis | Questionnaires submitted with an atypical timestamp | Were rapid responses excluded? | Not applicable; all submissions completed within the normal time range. |
| Analysis | Statistical correction | Were any statistical adjustments made? | No weighting applied; data used as descriptive, exploratory pilot findings. |
