# Supplementary figures and images for: Online Discussions of Men’s Mental Health on Reddit and YouTube: Cross-Sectional Mixed-Methods Infodemiological Study
Source: JMIR Infodemiology. 2026 May 5;6:e81315. doi: 10.2196/81315 (PMC13187704; doi:10.2196/81315)

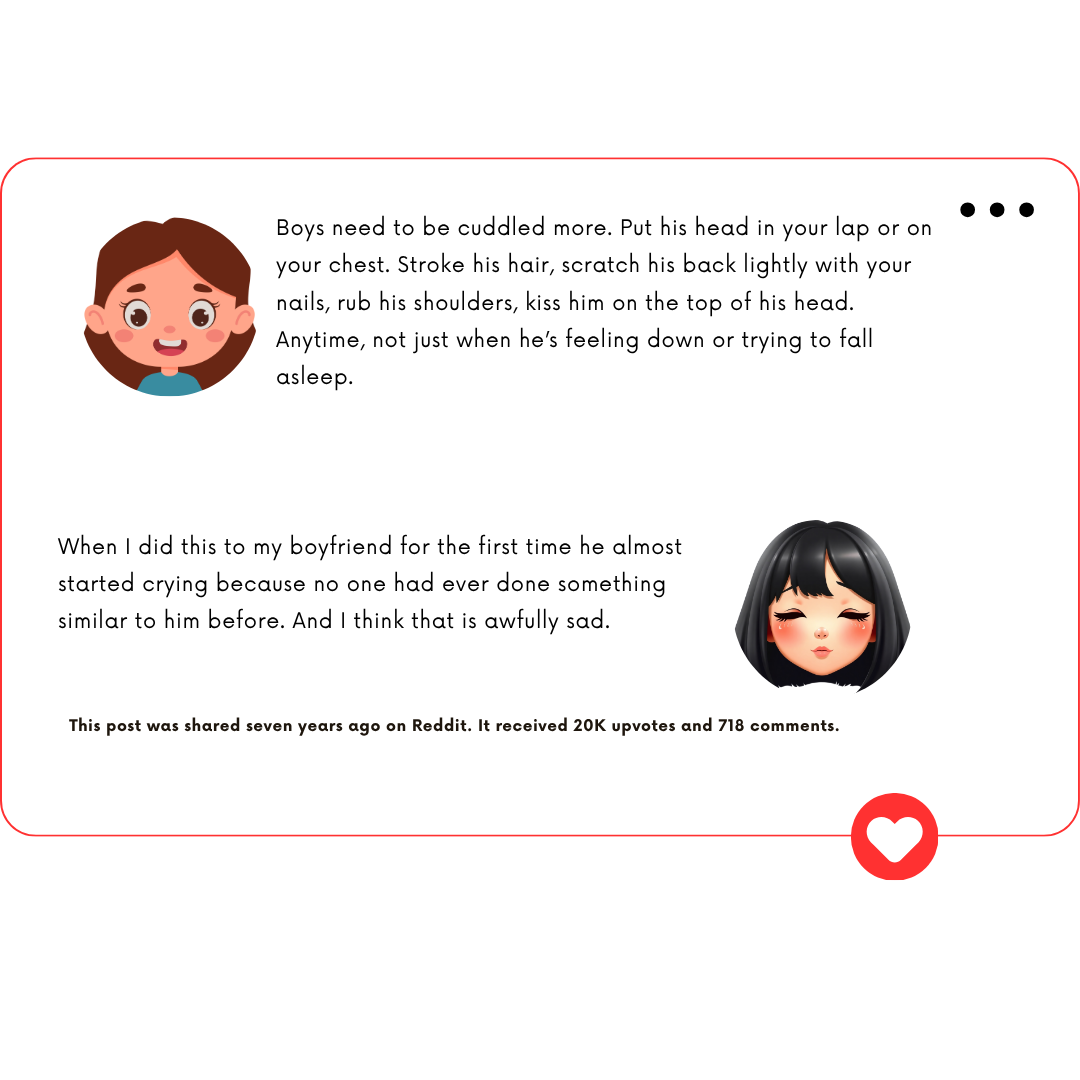

Supplement: Multimedia Appendix 2 [file infodemiology_v6i1e81315_app2.png]

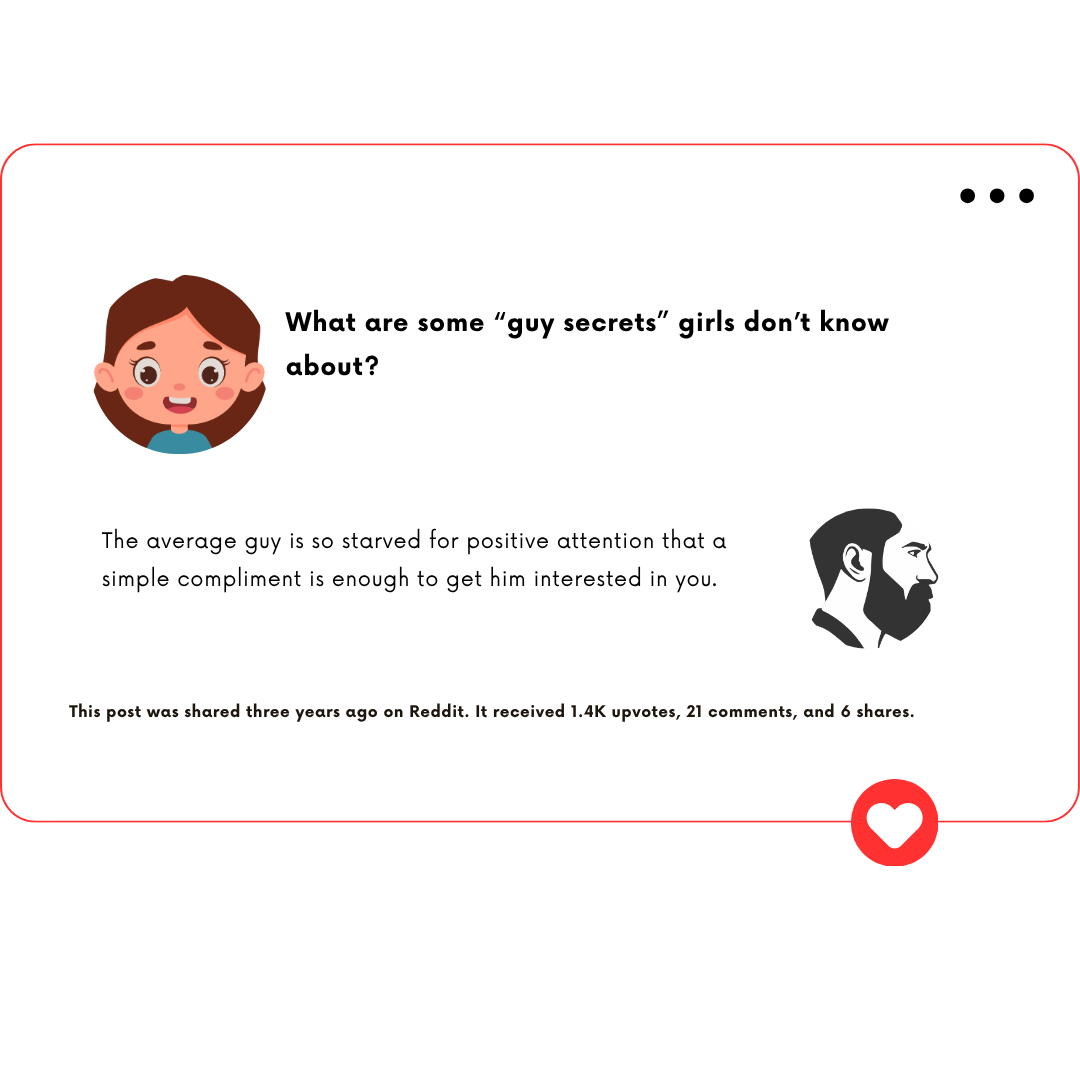

Supplement: Multimedia Appendix 3 [file infodemiology_v6i1e81315_app3.png]

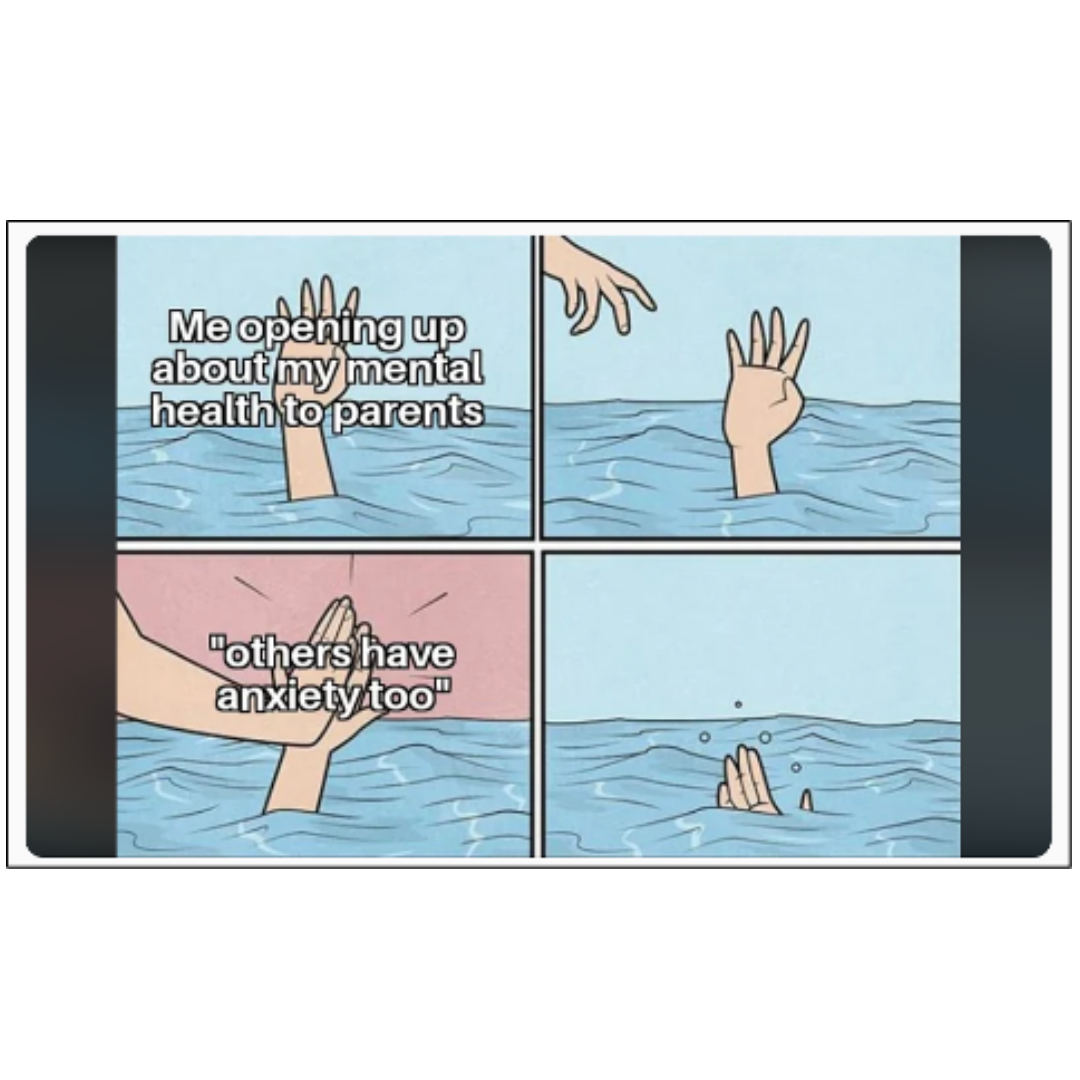

Supplement: Multimedia Appendix 4 [file infodemiology_v6i1e81315_app4.png]

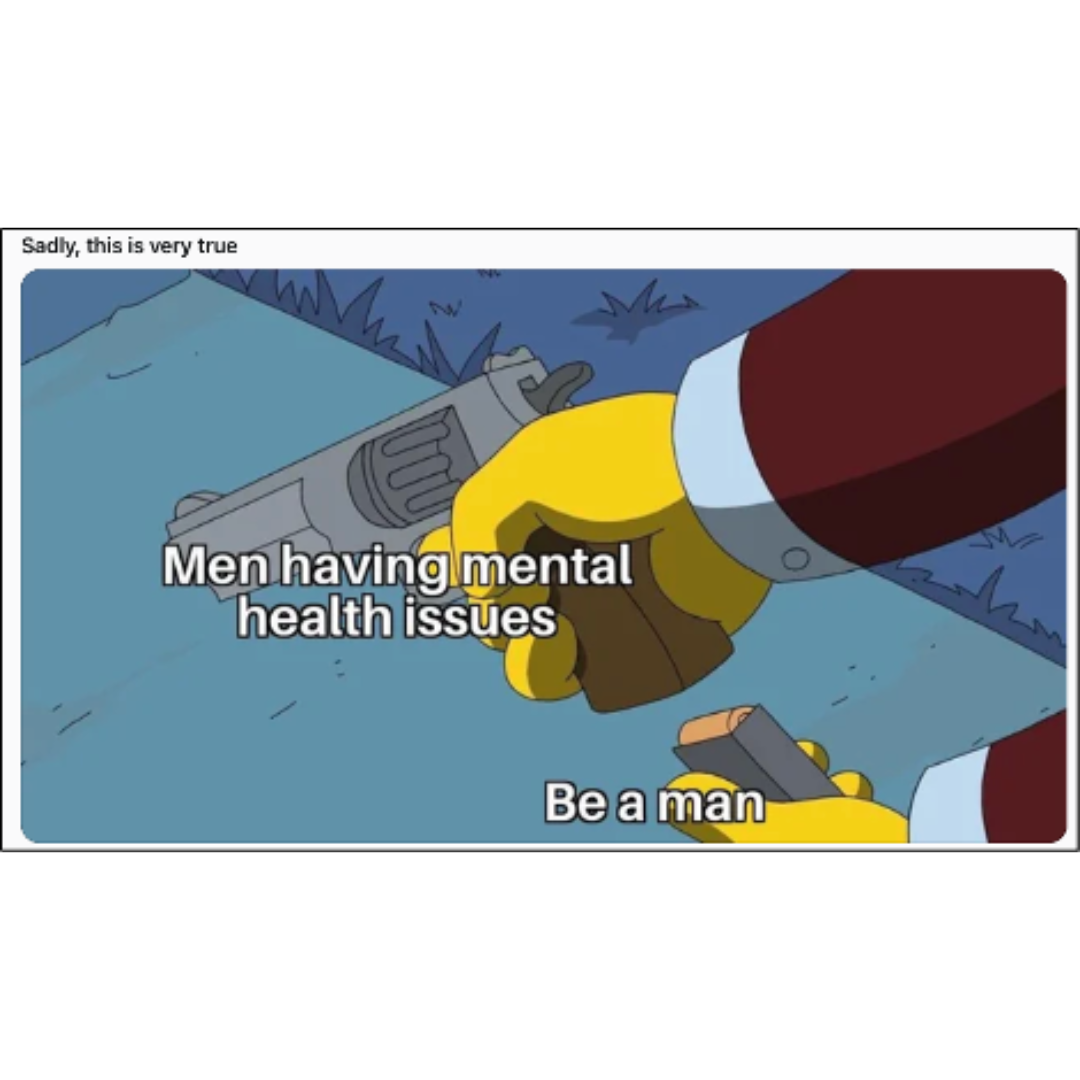

Supplement: Multimedia Appendix 5 [file infodemiology_v6i1e81315_app5.png]

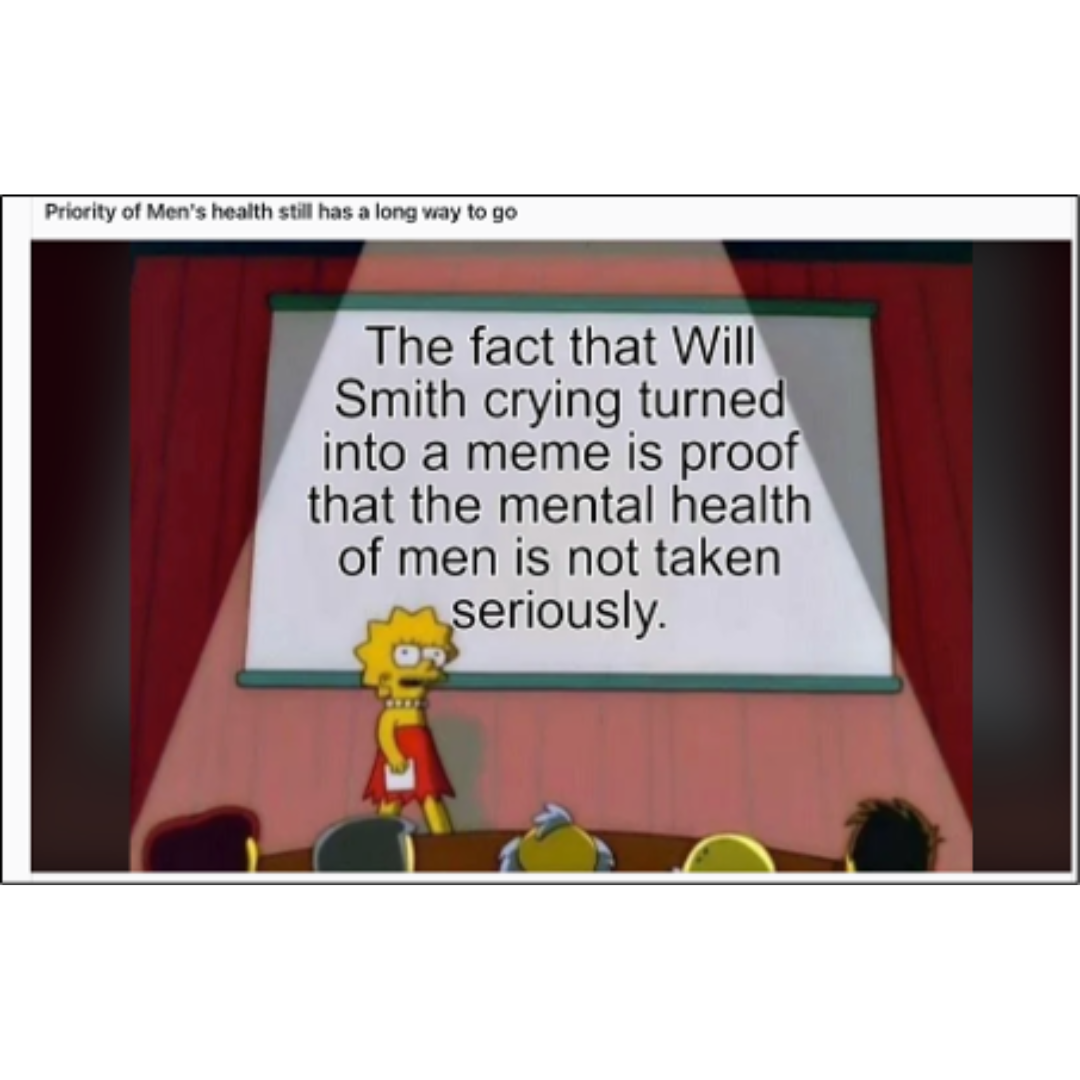

Supplement: Multimedia Appendix 6 [file infodemiology_v6i1e81315_app6.png]

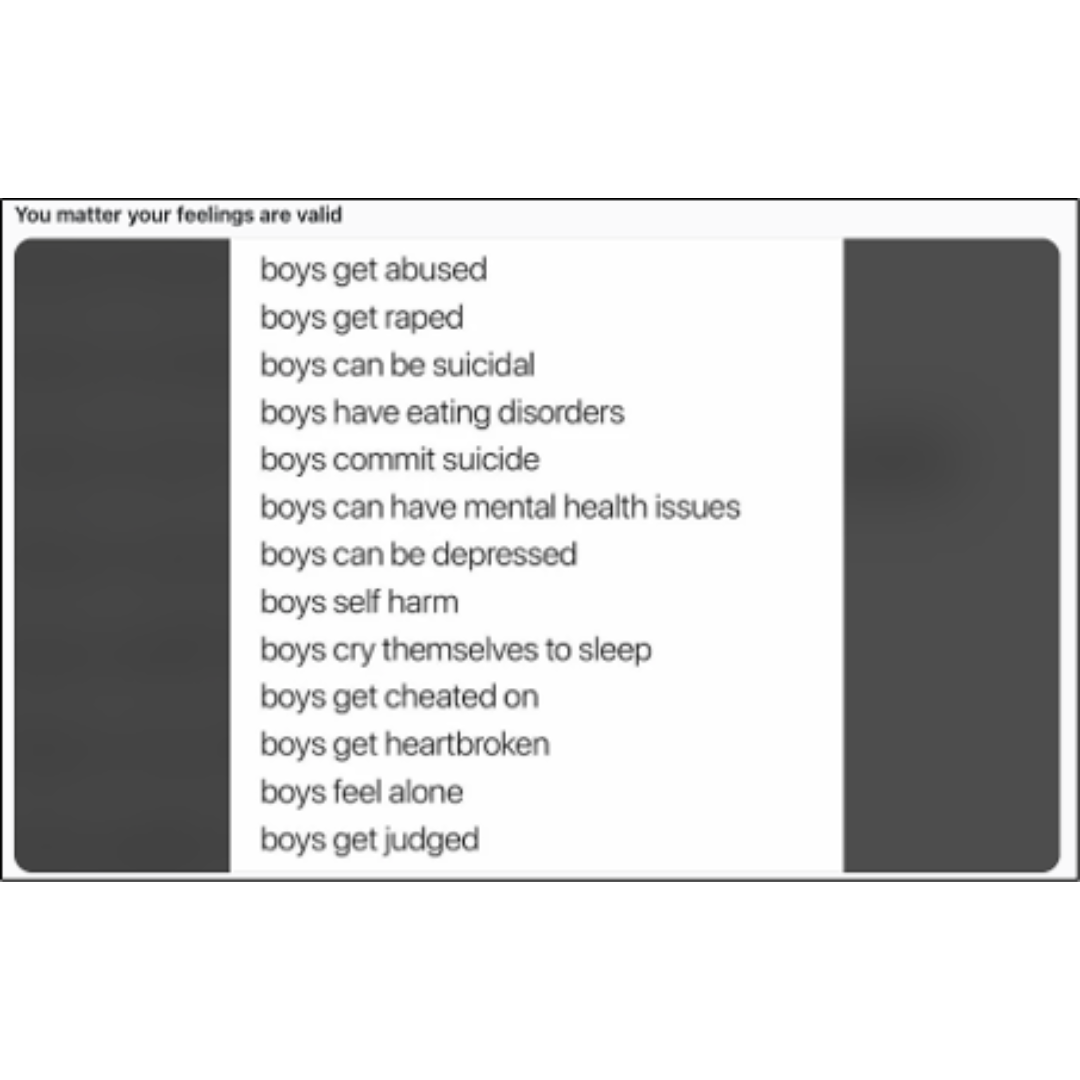

Supplement: Multimedia Appendix 7 [file infodemiology_v6i1e81315_app7.png]

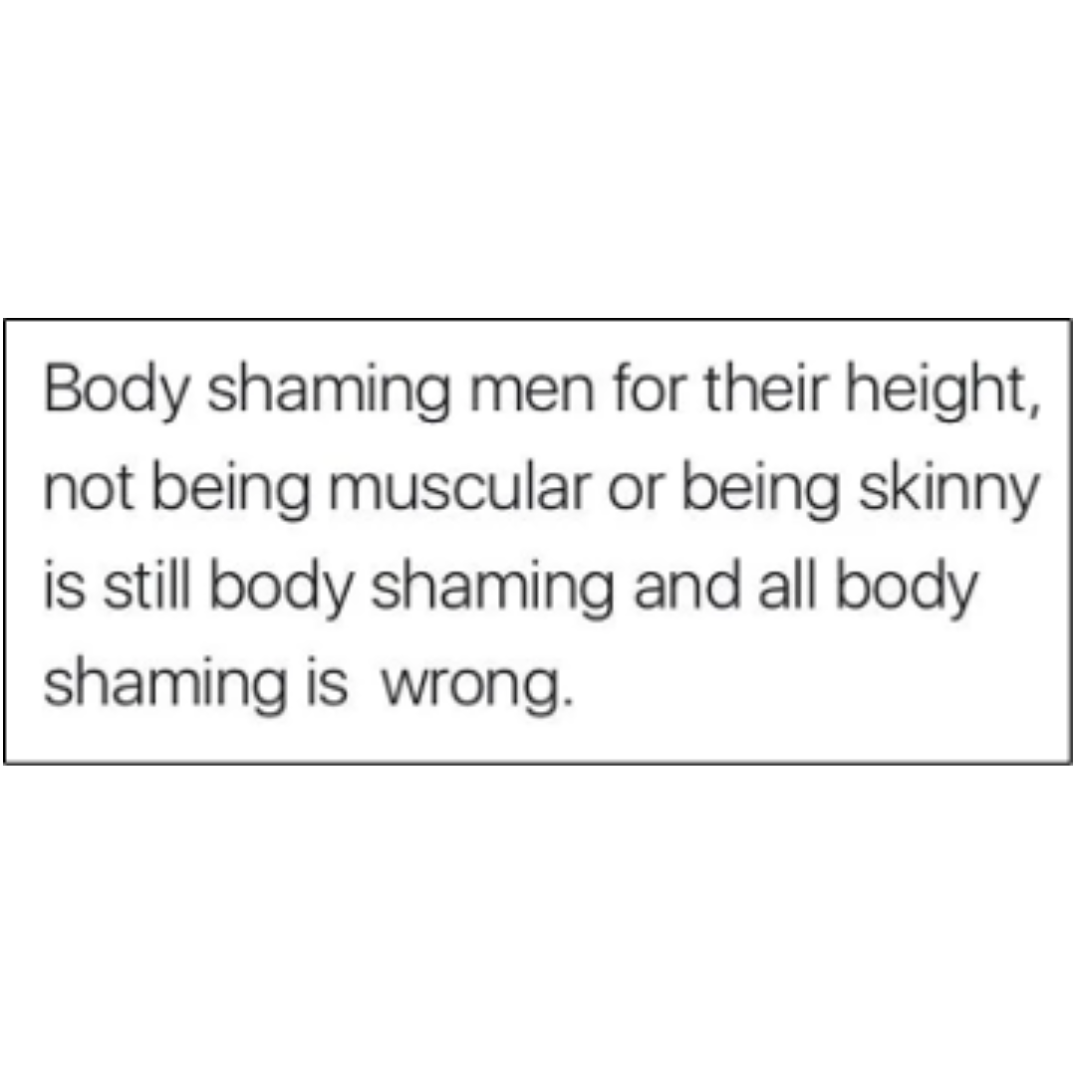

Supplement: Multimedia Appendix 8 [file infodemiology_v6i1e81315_app8.png]

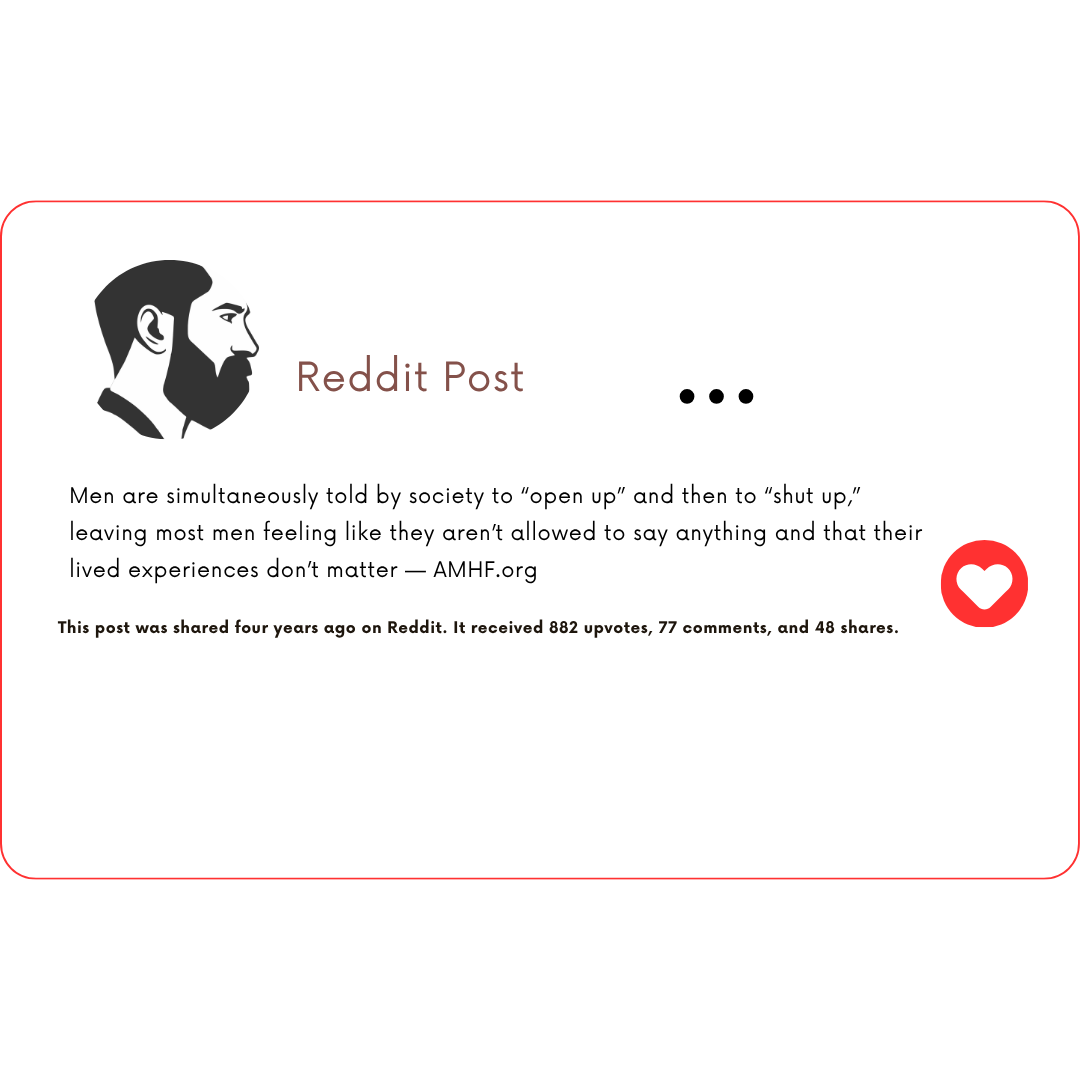

Supplement: Multimedia Appendix 9 [file infodemiology_v6i1e81315_app9.png]

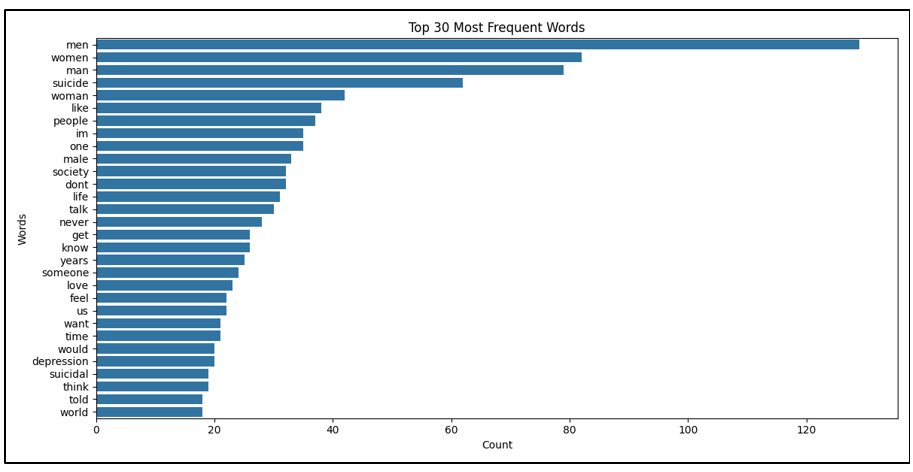

Supplement: Multimedia Appendix 10 [file infodemiology_v6i1e81315_app10.png]

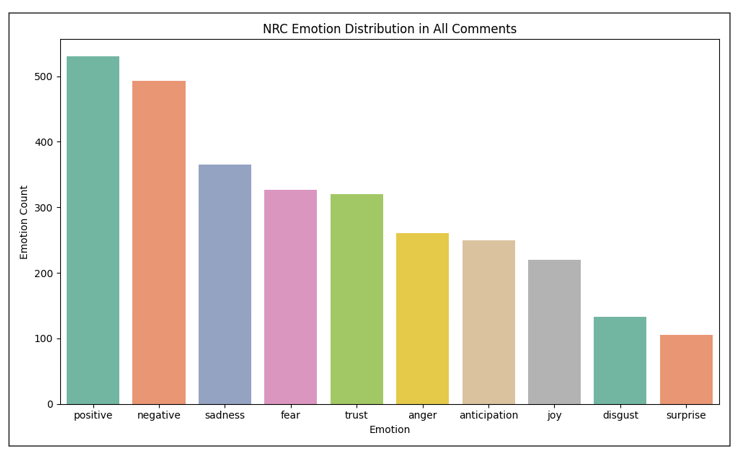

Supplement: Multimedia Appendix 11 [file infodemiology_v6i1e81315_app11.png]
